# *In Silico* Method for the Screening of Phytochemicals against Methicillin-Resistant *Staphylococcus Aureus*

**DOI:** 10.1155/2023/5100400

**Published:** 2023-05-18

**Authors:** Riaz Tabassum, Sumaira Kousar, Ghulam Mustafa, Amer Jamil, Syed Awais Attique

**Affiliations:** ^1^Department of Biochemistry, University of Agriculture, Faisalabad 38040, Pakistan; ^2^Department of Biochemistry, Government College Women University, Faisalabad, Pakistan; ^3^Department of Biochemistry, Government College University, Faisalabad 38000, Pakistan; ^4^School of Interdisciplinary Engineering & Science (SINES), National University of Sciences & Technology (NUST), Islamabad, Pakistan; ^5^Agency for Science, Technology and Research (A^∗^STAR), Bioinformatics Institute, 30 Biopolis Street, Matrix, Singapore 138671, Singapore

## Abstract

Methicillin-resistant *Staphylococcus aureus* (MRSA) has evolved resistance even against the last resort *β*-lactam antibiotics. This is because of the acquisition of an additional penicillin-binding protein 2a (PBP2a) which is a resistance determinant in MRSA. Currently, available PBP2a inhibitors are ineffective against life-threatening and fatal infections caused by microorganisms. Therefore, there is an urgent need to screen natural compounds that could overpass the resistance issue alone or in combination with antibacterial drugs. We studied the interactions of different phytochemicals with PBP2a so that crosslinking of peptidoglycans could be inhibited. In structure-based drug designing, *in silico* approach plays a key role in determining phytochemical interactions with PBP2a. In this study, a total of 284 antimicrobial phytochemicals were screened using the molecular docking approach. The binding affinity of methicillin, -11.241 kcal/mol, was used as the threshold value. The phytochemicals having binding affinities with PBP2a stronger than methicillin were identified, and the drug-likeness properties and toxicities of the screened phytochemicals were calculated. Out of the multiple phytochemicals screened, nine were found as good inhibitors to be PBP2a, among which cyanidin, tetrandrine, cyclomorusin, lipomycin, and morusin showed strong binding potential with the receptor protein. These best-selected phytochemicals were also docked to the allosteric site of PBP2a, and most of the compounds revealed strong interactions with the allosteric site. These compounds were safe to be used as drugs because they did not show any toxicity and had good bioactivity scores. Cyanidin had the highest binding affinity (S-score of -16.061 kcal/mol) with PBP2a and with high gastrointestinal (GI) absorption. Our findings suggest that cyanidin can be used as a drug against MRSA infection either in purified form or that its structure can lead to the development of more potent anti-MRSA medicines. However, experimental studies are required to evaluate the inhibitory potential of these phytochemicals against MRSA.

## 1. Introduction

The Gram-positive *Staphylococcus aureus* is a foremost human pathogen that causes an extensive range of clinical infections and diseases [1]. *S. aureus* has been identified as the most common agent which is responsible for infections related to soft tissues and skin and causes skin abscesses, furuncles, and carbuncles. Skin and soft tissue infections are frequently initiated as abscesses or minor boils and can cause severe muscle or bone infections. They can also disseminate to the heart valves (i.e., endocarditis) or lungs. *S. aureus* is a significant cause of food-borne sickness, causing an estimated 241,000 illnesses per year in the United States [2]. The incidence rate of MRSA infection per year has been found to be 20 to 50 cases/100,000 population with 10-30% mortality rate. Comparatively, these infections account for more number of deaths than for AIDS, viral hepatitis, and tuberculosis combined [3].

The bacterial cell wall is a polymer of peptidoglycans as its principal building blocks, and therefore, the health of the cell wall is responsible for bacterial survival during cell division and growth. Peptidoglycan is made up of repeating units of the disaccharide N-acetylglucosamine (NAG)-N-acetylmuramic acid (NAM) with peptide stems on the NAM unit. Crosslinking of peptidoglycans is done by penicillin-binding proteins (PBPs) through their transpeptidase domain [4]. PBPs are desirable targets for antibiotics, especially for *β*-lactams due to their key role in bacterial survival. Four types of PBPs, designated as PBP1-PBP4, have been identified in *S. aureus*. *β*-Lactams backbone has a structural similarity with the peptide stem of peptidoglycan. *β*-Lactam antibiotics bind to the transpeptidase domain of PBPs, and crosslinking of peptidoglycans is inhibited [5, 6]. Hence, *β*-lactams can efficiently inhibit the PBPs and clear the bacterial infection.

Along with the native PBPs, there is another low-affinity penicillin-binding protein known as PBP2a which is a resistance determinant in MRSA. PBP2a is encoded by the methicillin resistance A *(mecA)* gene. The *Staphylococcal* Chromosome Cassette mec (SCCmec) of 21 to 60 kb contains the *mecA* gene. The SCCmec is a moveable genetic component that may also hold genetic structures such as pUB110, Tn554, and pT181 which encode resistance to non-*β*-lactam antibiotics. Mostly MRSA and other methicillin-resistant *Staphylococci*, defined to date, harbor the *mecA* gene present in association with SCCmec types I–XI and their subtypes [7]. Owing to the presence of *mec* genes such as *mecA, mecB,* and *mecC,* which code for specific PBPs, MRSA expresses resistance to *β*-lactam antibiotics [8]. PBP2a also plays a significant role in vancomycin-intermediate *S. aureus*-type glycopeptide resistance. From the *vanA* gene complex in vancomycin-resistant *S. aureus*, PBP2a is necessary to express high-level vancomycin resistance [9].

The active site of PBP2a exists in a closed conformation deep in a tight groove; hence, it is inaccessible to inhibition by *β*-lactam antibiotics. In order to carry out the transpeptidation reaction, it opens to accommodate the two peptidoglycan strands in cell wall synthesis. It requires an active site with a volume of more than 1000 Å^3^ that is larger compared to the one which is needed for the interaction with an antibiotic. Protein shows conformational changes by allosteric modulation. Two major grooves are present in PBP2a (i.e., one at the active site, where peptidoglycan or antibiotic binds, and the other is an allosteric domain that is found at a distance of 60 Å from the active site), and both sites are involved in catalysis. The binding at the allosteric domain can trigger a conformational change that activates the protein for the transpeptidase activity [6].

Antibiotics have not only increased bacterial resistance due to the evolutionary adaptation of microorganisms but also have harmful side effects [10]. Severe side effects of antibiotics include mitochondrial toxicity, Stevens-Johnson syndrome, aminoglycoside-induced cytotoxicity, hypersensitivity reactions resulting in anaphylaxis, and fatal hepatic necrosis [11].

Chemical substances which have antimicrobial properties and determine the medicinal value of plants are known as phytochemicals [12]. Phytochemicals are structurally classified into alkaloids, flavonoids, isoflavones, flavan-3-ols, anthocyanins, carotenoids, triterpenoids, coumestans, hydroxycinnamic acids, phenolic acids, lignans, monophenols, monoterpenes, organosulfides, phytosterols, saponins, stylbenes, and xanthophylls. Flavonoids are the most extensively studied compounds and are reported to have a wide range of pharmacological activities [13]. Similarly, phenolic compounds are efficient against chronic disease, cardiovascular diseases, and different cancers [14]. The capacity of phenolic compounds to eliminate reactive oxygen species while avoiding the initiation of additional oxidative reactions is well documented [15]. Currently, the popularity of *in silico* methods is increasing rapidly due to their implementation and applications in medical science. In molecular docking, different ligand conformations are compiled in the selected active sites of receptor proteins, and then the best binding conformations are ranked according to their binding conformation energies [13].

Considering the role of PBP2a in resistance development in *S. aureus* against *β*-lactams, finding active phytochemicals which could block PBP2a activity is an essential step towards the development of anti-MRSA drugs. In the present study, we analyzed interactions of different phytochemicals with active and allosteric sites of PBP2a using MOE 2011.10 software and evaluated their potential against MRSA [16].

## 2. Materials and Methods

### 2.1. PBP2a Receptor Protein Acquisition

Penicillin-binding protein 2a (PBP2a) from MRSA was searched in the RCSB PDB database. PBP2a protein with the highest resolution of 1.5 Å in complex with quinazolinone ligand (PDB ID =4CJN) was downloaded as a PDB file [17].

### 2.2. Physiochemical Properties of PBP2a

The Pepstats database [18] and the ProtParam tool from the ExPASy database [19] were used to predict the physicochemical properties of PBP2a.

### 2.3. Secondary Structure Prediction

The PDBsum database was used to predict the secondary structure of the PBP2a protein [18].

### 2.4. PBP2a Protein Preparation as a Receptor

PBP2a was opened in molecular operating environment (MOE) 2011.10 software with default parameters. PBP2a is a homodimer protein with two similar chains. Quinazolinone ligand and one chain were deleted from the PDB structure using the sequence editor window in MOE. Water molecules were also deleted to avoid solvent-mediated salt bridge interactions of ligands with receptor active and allosteric sites. Hydrogens were added to the receptor protein using the PROTONATE 3D function with default parameters in MOE. The energy of the receptor was minimized by the energy minimize function using the MMFF94X Forcefield algorithm. Active and allosteric sites of PBP2a were identified by the Site Finder tool in MOE software. Active sites falling within the transpeptidase domain of PBP2a as reported in the UniProt database (https://www.uniprot.org/) were selected, and dummy atoms of these sites were created [20]. Each ligand could search only for these dummy atoms. The prepared PBP2a receptor molecule was saved as .moe file format.

### 2.5. Ligands Acquisition and Preparation for Docking

Antimicrobial plant phytochemicals were searched from literature, the *ChemSpider database (*http://www.chemspider.com/), ZINC database (https://zinc.docking.org/), ChemFaces database (http://www.chemfaces.com/), and PubChem database (https://pubchem.ncbi.nlm.nih.gov/). 3D structures of ligands were downloaded in the SDF file format. Phytochemicals whose 3D structures were not reported in the databases were built using the CHIMERA software and Builder window in MOE software and saved in mol2 file format. A database of these 284 plant phytochemicals was made. Hydrogens were added, and energy was minimized by PROTONATE 3D function and MMFF94X Forcefield algorithm, respectively. This database was saved as a plant phytochemicals database. Two-dimensional structures of the top 9 phytochemicals were computed using the 2D Molecule function in MOE software.

### 2.6. Penicillins and General Antibiotics Preparation for Docking

Twelve classes of penicillin antibiotics were searched in the PubChem database, and the 3D structures of these penicillins were downloaded in SDF file format. A separate database for these penicillins was made. Hydrogens were added, and the energy was minimized by the PROTONATE 3D function and MMFF94X Forcefield algorithm, respectively, as mentioned above. This database was saved as the Penicillins Antibiotics database. Other than penicillin, 9 general types of antibiotics were searched and the 3D structures of these antibiotics were downloaded in SDF file format. A separate database for these different antibiotics was made. Hydrogens were added and energy was minimized by PROTONATE 3D function and MMFF94X Forcefield algorithm, respectively, as mentioned above. This database was named as Different Classes of Antibiotics database and saved in .mdb format. The 2D structures of penicillins and 9 general antibiotics were computed using the 2D Molecule Function in MOE software.

### 2.7. Molecular Docking

Docking was performed using MOE software. MOE was selected for molecular docking because of its better graphical user interface. Ligand position and interactions with receptor residues are better visualized in MOE. It has multidisciplinary applications such as medicinal chemistry applications, structure-based design, biologics applications, pharmacophore discovery, fragment-based design, molecular modelling, molecular dynamics simulation, protein and antibody modeling, cheminformatics, and QSAR models for drug discovery. It also identifies hydrogen bonds, hydrophobic interactions, salt bridges, cation-*π*, sulfur-LP, and solvent exposure [21]. Molecular docking was performed by selecting the docking parameters as follows: the ligand placement method was selected as a triangular matcher algorithm for the top 1000 poses of the docked molecule. Generated poses were rescored by the London dG scoring function. The force field refinement algorithm with 500 iterations was selected for further refinement of the top 10 ranked poses generated by the London dG scoring function. The generalized Born solvation model (GBVI/WSA dG) was selected for final binding energy calculation while keeping receptor residues rigid. The S-score or binding score indicates the binding affinities of ligands with the receptor in kcal/mol. S-score was used for the ranking of top binding affinities of ligands with the receptor active sites. Dummy atoms of selected active sites and allosteric site of PBP2a were selected as a receptor. Databases of plant phytochemicals, penicillin antibiotics, and different classes of antibiotics were docked with dummy atoms by selecting the same parameters mentioned above [22]. The most negative the S-score, the strongest the binding of the ligand with the receptor residues. Output results were sorted according to S-score. Methicillin S-score was set as a threshold score for screening plant phytochemicals for further analysis. 2D structures of phytochemicals and antibiotics were computed using 2D molecule function in MOE.

### 2.8. Toxicities and Drug Likeliness Properties Prediction of Phytochemicals

ADME (i.e., absorption, distribution, metabolism, and excretion) are important parameters of a drug in pharmacokinetics and pharmacology. The ADME properties of molecules depend upon Lipinski's rule of five (Ro5) which is defined as: the molecular mass of a compound should not be greater than 500 g/mol; the number of hydrogen bond acceptor (HBA), and number of hydrogen bond donor (HBD) should not be greater than 10 and 5, respectively, and lipophilicity (Log*P*) should be less than 5. Less than two violations of these rules are acceptable for good drug properties [23, 24]. The SwissADME database was used to predict the ADME properties and other drug-likeness properties of small molecules. Canonical smiles of phytochemicals were uploaded to the SwissADME database. Results from the SwissADME database were downloaded as a CSV file [25]. ADME properties were also calculated for penicillin antibiotics and 9 general types of antibiotics. The results were downloaded as a CSV file. Phytochemicals were further investigated for their toxicities using DataWarrior 5.0.0 software. Toxicities were predicted as mutagenic, tumorigenic, reproductively effective, and irritant [26]. The bioactivities of phytochemicals were predicted using the Molinspiration database online (http://www.molinspiration.com/). The bioactivities of the compounds were predicted as GPCR ligand, kinase inhibitor, protease inhibitor, enzyme inhibitor, nuclear receptor ligand, and ion channel modulator.

### 2.9. Ligand Interactions

Interactions of the ligand with the receptor were computed using the ligand interaction function in MOE software. Ligand interactions in the 2D graph indicate the hydrogen bonding, hydrophobic interactions, Van der Waals forces, and electrostatic interactions of ligands within the active sites of PBP2a [27]. Three-dimensional poses of ligands with PBP2a were created using PyMol Molecular Graphics software 2.2.3 version. 2D and 3D ligand interactions of the top 5 plant phytochemicals, penicillins, including methicillin, and other general antibiotics to the active sites of the PBP2a were generated. The interactions of the top 9 plant phytochemicals with the allosteric site were also generated.

## 3. Results

### 3.1. 3D Structure and Physicochemical Properties of PBP2a

**Figure 1:**
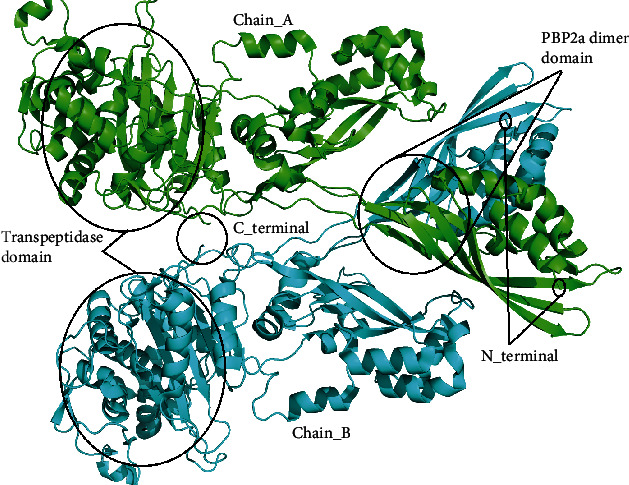
3D-structure of PBP2a (PDB ID: 4CJN) visualized in PyMol. PBP2a is homo dimer of chain A and chain B.

Functional PBP2a is a homodimer protein, and each chain has 642 amino acids. Three domains have been reported in this protein: the 2-114 amino acid sequence is the NTF2-like N-terminal transpeptidase domain, the 122-283 amino acid sequence length is the penicillin-binding protein dimerization domain, and the 320-631 amino acid sequence length is the penicillin-binding protein transpeptidase domain of PBP2a [20]. Both chains are highlighted in different colors in the PyMol software (Figure 1).

Table 1 demonstrates the physicochemical properties of PBP2a. The PBP2a had extinction coefficient value of 90650 M^−1^ cm^−1^ at 280 nm. An aliphatic index of a protein is the relative volume occupied by aliphatic side chains (i.e., alanine, isoleucine, leucine, and valine). Aliphatic index of 78.97 indicates the thermostability of PBP2a. Instability Index (II) below 40 is considered stable [28], so the II value of 31.30 is showing PBP2a as stable. The GRAVY value for a peptide or protein is the sum of hydropathic values of all the residues divided by the total number of amino acids in the sequence. The prediction of the GRAVY value of PBP2a as -0.802 shows that it is hydrophilic.

### 3.2. Secondary Structure Analysis

**Figure 2:**
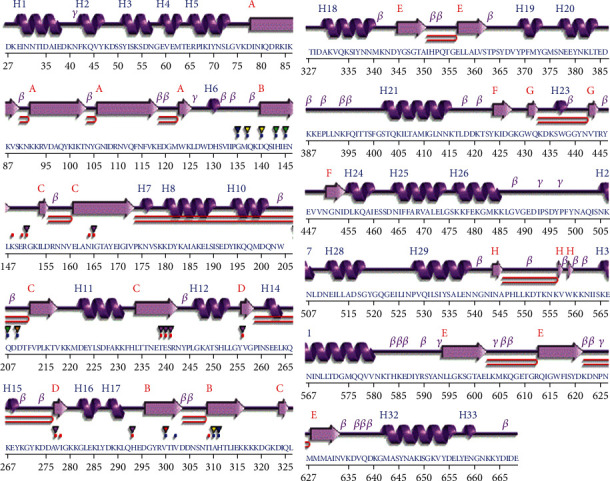
Secondary structure predicted by the PDBsum database. Helices labelled H1, H2,...Hn and strands by their sheets (A, B). “*β*” indicates beta turn, “*γ*” indicates gamma turn, and “===” indicates beta hairpin. The active-site serine (Ser403) at the N-terminus of the H21 helix in the sequence motif SXXK contributes to the manifestation of resistance in MRSA.

The secondary structure of the PBP2a protein was also predicted. The architecture and the folding pattern of most of the proteins are supported by their secondary structures, so an accurate assignment for the prediction of secondary structure elements (i.e., *α*-helices and *β*-sheets) is an important problem [29]. The prediction of the secondary structure of proteins is also the key to the generation of schematic diagrams of their 3D structures because it simplifies the complex atom-level description of proteins [30]. The active-site serine (Ser403) at the N-terminus of the H21 helix in the sequence motif SXXK contributes to the manifestation of resistance in MRSA (Figure 2) [31].

### 3.3. Active Sites Identified in PBP2a for Docking

Active sites of PBP2a were identified, as shown in Table 2. Only those sites were selected for docking which contained the maximum number of residues of the transpeptidase domain. Site1 had the largest size and was composed of the highest number of residues. Ser403 amino acid, a resistance determinant in MRSA, was present in Site1. The size column indicates the number of alpha spheres comprising the site. The PLB column indicates the propensity for ligand binding score for the contact residues in the receptor. The Hyd column indicates the number of hydrophobic contact atoms in the receptor. The side column indicates the number of sidechain contact atoms in the receptor. The residues column indicates the residues that make up the active site. The list is sorted by the PLB column (in descending order).

### 3.4. Docking Output Results

Different conformational poses of each ligand against PBP2a were computed in MOE software. Only the conformation pose of a ligand with PBP2a was selected that showed the strongest binding affinity (S-score in kcal/mol) and had a root-mean-square deviation (RMSD) score below 2. However, binding affinity is not the only factor for the determination of the inhibition potential of phytochemicals. Molecular interactions and RMSD values are also key determinants for the inhibition potential of ligands. In the docking simulation, the RMSD value determines the quality of ligand conformation. A RMSD value of a ligand pose below 2 Å is categorized as a good and acceptable binding pose of a ligand with a receptor. The pose of a ligand with an RMSD value above 3 Å is unacceptable ligand conformation [16]. The compound N-acetyl-muramic acid was also docked to the active site (site1) of PBP2a, which showed substantial binding potential to the receptor protein. It exhibited an S-score of -13.40 kcal/mol and an RMSD value of 0.997 (Figure S15), which is comparable to the best-selected phytochemicals docked to different active sites of PBP2a.

### 3.5. Phytochemicals Filtering by Lipinski's Ro5 and DataWarrior Software

**Figure 3:**
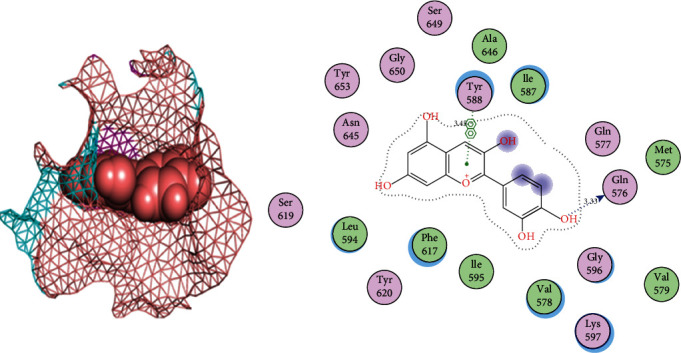
2D and 3D interactions of cyanidin with site3 of PBP2a. Mesh indicates the site3 and balls indicates the cyanidin. Cyanidin makes two bonds with Tyr588 (bond length = 3.45) and Gln576 (bond length = 3.33).

**Figure 4:**
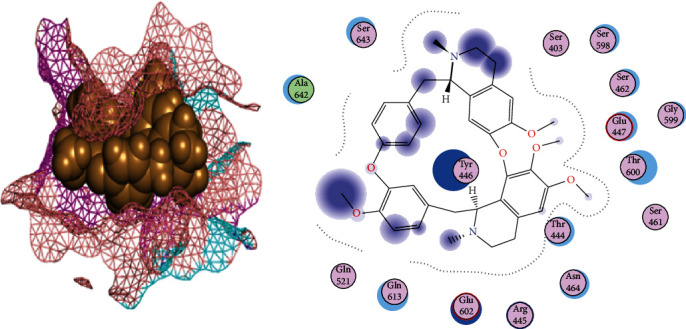
2D and 3D interactions of tetrandrine with site1 of PBP2a.

**Figure 5:**
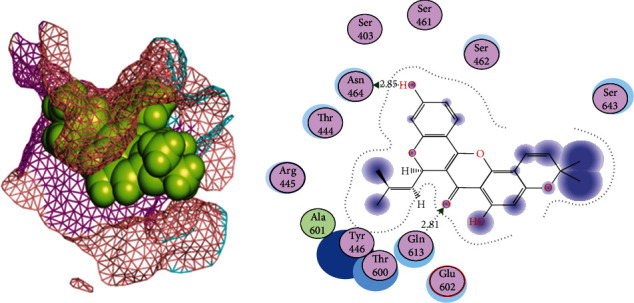
2D and 3D interactions of cyclomorusin with site1 of PBP2a.

**Figure 6:**
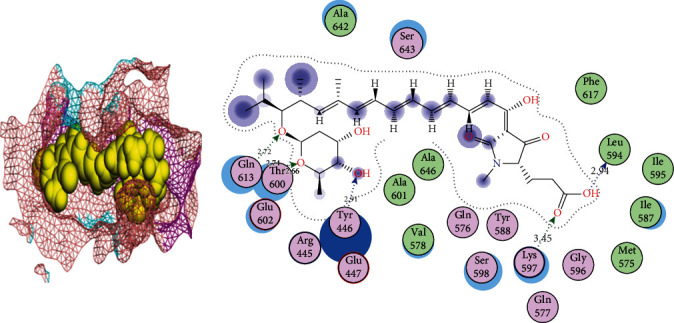
2D and 3D interactions of lipomycin with site1 of PBP2a.

**Figure 7:**
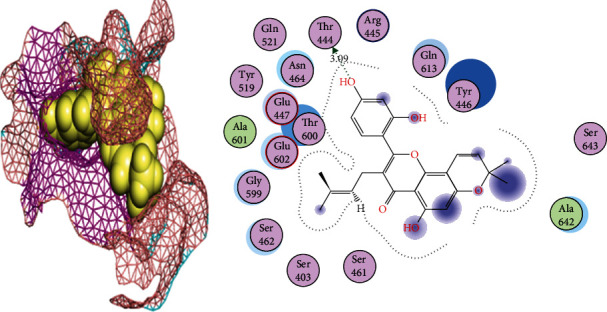
2D and 3D interactions of morusin with site1 of PBP2a.

Out of 284 antimicrobial plant phytochemicals, 119 satisfied Lipinski's Ro5. As we focused on *methicillin-resistant Staphylococcus aureus* (MRSA) in this study, the methicillin S-score was taken as a threshold score, i.e., -11.241 kcal/mol. Fifteen phytochemicals crossed the threshold S-score limit of -11.241 kcal/mol. Plant phytochemicals which did not cross this threshold value are meaningless in this study. The toxicities of these phytochemicals were then evaluated by DataWarrior software. Phytochemicals that showed high mutagenic, tumorigenic, irritant, and reproductive-effect properties were also eliminated. Only 9 phytochemicals were screened, which did not show any toxicities. The bioactivity scores of the 9 phytochemicals against different receptor enzymes were predicted by the Molinspiration database. Two-dimensional and three-dimensional interaction diagrams of the top five strong inhibitors of PBP2a are shown in (Figures 3–7).

### 3.6. Phytochemicals That Follow Ro5 and Do Not Cause Toxicities

Our docking studies screened the top 9 phytochemicals that fulfilled the Lipinski criteria, and the S-scores of these compounds were above the threshold score of -11.241 kcal/mol. Cyanidin had the strongest binding affinity at -16.06 kcal/mol with RMSD =1.42 Å, indicating that it is a strong inhibitor of PBP2a in MRSA. It makes bond with site3 residues Gln576 (bond length = 3.33) and Tyr588 (bond length = 3.46) of the PBP2a protein (Figure 3). Spheres indicate the cyanidin ligand and mesh indicate the binding site3 of PBP2a in the 3D interactions figure. The cyaniding, therefore, can be used for the treatment of MRSA infections with high recovery rates from infections. All inhibitors except lipomycin and rosmarinic acid had high gastrointestinal (GI) absorption which makes them strong drug candidates with strong binding affinities with PBP2a compared to different classes of penicillin antibiotics (Table 3).

### 3.7. Molecular Docking to the Allosteric Site of PBP2a

The residues of the allosteric site of PBP2a are also given in Table 2. The site was predicted by MOE software and used for molecular docking study. The 2D and 3D interactions of the top 9 ligands have been shown in Figures S16-S24 of supplementary file. The binding scores (S-scores in kcal/mol) and the RMSD values are given in Table 4. The ligand morusin with best S-score of -14.843 kcal/mol showed interactions with Asp275, Val277, and Glu294 amino acids of the allosteric site. Cyanidin with binding score of -13.199 kcal/mol interacted with Asp275. Cyclomorusin and lipomycin also exhibited strong interactions with the allosteric site residues (i.e., Ala276 and Asp295 with S-score of -12.013 kcal/mol and Gln292 and Lys322 with S-score of -12.819 kcal/mol, respectively). All ligands exhibited better binding scores and interactions with the residues of the active site compared to the allosteric site of PBP2a except morusin, which showed a binding score of -13.755 kcal/mol with the binding site and -14.843 with the allosteric site.

The top 9 plant phytochemicals with their names, PubChem-CIDs, 2D structures, binding affinities, RMSD scores, and the binding sites of PBP2a where these inhibitors could bind are shown in Table 4. The ADME properties and toxicities and bioactivities of these compounds are shown in Tables 3 and 5, respectively.

### 3.8. Phytochemicals That Did Not Follow Ro5

In our findings, some phytochemicals had a high binding score, even more than any type of antibiotics used, in this study but did not follow the Ro5. Names, PubChem IDs, 2D structures, binding scores, RMSD values, and binding sites of the receptor with which these ligands were bound are shown in Table S1. ADME properties and toxicities and bioactivities of these compounds are shown in Table S2 and Table S3, respectively. 2D and 3D interactions of the top 5 phytochemicals which did not follow the Lipinski's test are shown in figures S1-S4. In spite of poor GI absorption, adverse GI effects, and potential toxicity and nephrotoxicity, these drugs are being used by physicians to cure acute infections. In our docking study for screening the potent anti-MRSA plant phytochemicals, we evaluated the phytochemicals having high binding affinities compared to the general antibiotics such as amikacin, gentamicin, vancomycin, and oxytetracycline. Asphodoside-D, bacopasaponin A, and diosmin phytochemicals had binding affinities greater than all types of antibiotics used in this study. The 2D and 3D interactions of some of the penicillins and general antibiotics with their respective site(s) are shown in figures S5-S14.

### 3.9. Penicillin Antibiotics Docking Results

Different types of penicillin antibiotics along with the methicillin antibiotic were docked as a reference for the screening of the potent inhibitors of PBP2a protein. Ticarcillin showed the highest binding affinity (i.e., -13.753 kcal/mol) and was bound to the site1. Methicillin also bound to the site1 with a binding affinity score of -11.241 kcal/mol, and this score was used as a threshold score for the screening of phytochemicals (Table S4). Drug-likeness properties of antibiotics were also computed for comparison of drug-likeness properties of phytochemicals. Our finding indicates that piperacillin did not follow the Ro5. Ticarcillin, carbenicillin, carbenicillin, and dicloxacillin along with piperacillin showed low GI absorption (Table S5). Toxicities of penicillins are shown in Table S6. Regardless the antimicrobial testing results, MRSA should be considered resistant to all penicillins, carbapenems, cephems, and other beta-lactams such as ampicillin/sulbactam, amoxicillin/clavulanate, piperacillin/tazobactam, ticarcillin/clavulanate, and imipenem, as most of the isolates react weakly to *β*-lactam [32].

### 3.10. Docking Results of General Antibiotics

Other than penicillins, 9 different types of general antibiotics were also docked using the same receptor binding sites as dummy atoms. The binding affinities of two antibiotics, ciprofloxacin and fusidic acid, did not cross the threshold score (i.e., -11.241 kcal/mol). Cefoxitin, chloramphenicol, and trimethoprim-sulfmethoxazole had almost similar binding affinities as penicillins (Table S7). Although amikacin, gentamicin, vancomycin, and oxytetracycline had high binding affinities with PBP2a, which did not obey Lipinski's rule of 5 and had low GI absorption, they must be given (Table S8). The toxicities of these general antibiotics are shown in Table S9.

## 4. Discussion

The purpose of this study was to explore the active site for PBP2a and molecular docking to find potential antibacterial plant-based ligands and interactions between selected ligands and the receptor protein of MRSA. Computational studies were used to predict the interactions of various ligand molecules against the receptor molecules. Computer-aided drug discovery helps scientists estimate the binding interactions of different molecules prior to their productions in the laboratory [33]. MOE is a versatile computer-aided drug design platform to amalgamate protein/DNA/RNA modelling, molecular docking and modelling, peptide modelling, energy evaluation, antibody designing, fragment-based discovery, and 3D visualization [34].

In this study, a total of 284 ligands from various medicinal plants were docked against the PBP2a of MRSA. As methicillin is resistant in nature, the S-score of methicillin (i.e., -11.241 kcal/mol) was set as a threshold value, and 119 ligands were selected as they crossed this threshold value. Among the selected 119 ligands, only 9 ligands accomplished the criteria of being good drug molecules because they followed the Lipinski rule of 5 and showed no violation. On the basis of interactions and docking scores, cyanidin, tetrandrine, cyclomorusin, lipomycin, morusin, aromadendrin, rosmarinic acid, chrysoeriol, and *α*-lapachone were selected as the top nine phytochemicals.

Cyanidin with an S-score of -16.06 kcal/mol was selected as the top candidate, and it showed binding patterns with Gln576 and Tyr588 at site3 of PBP2a of MRSA. Cyanidin, or cyanidol, also known as 7-hydroxyflavonoids, is a type of anthocyanin which is present in high concentration in berries (e.g., blueberries, cranberries, bilberries, elderberries, raspberry seeds, and strawberries). It has anticancer, antioxidant, antitoxic, and anti-inflammation properties. It also has also a positive effect on memory and learning [35]. Cyanidin plays different roles such as chemopreventive, antioxidant, and neuroprotective agent [36].

Cyclomorusin is a class of flavones found in the root bark of *Morus alba* (white mulberry) and *Artocarpus altilis* (breadfruit). Its leaves, fruits, roots, and twigs have all been used in traditional Chinese medicine [37]. Morusin is an antioxidant and anticancer agent present in the root bark of *Morus alba* (mulberry) and other Morus species [38]. Our findings indicate that Morus species, also known as mulberries, contain phytochemicals that are strong inhibitors of the PBP2a protein, and therefore, these plants can be used as a remedy to clear MRSA infections.

Tetrandrine is a bis-benzylisoquinoline alkaloid that has been extracted from the roots of the Chinese herb *Radix Stephania tetrandrae S. Moore*. It has been used as a remedy for arthritis and neuralgia in traditional Chinese medicine and also has antifungal properties. The biological activity of tetrandrine has been proven via its potential in various signalling pathways, caspase pathways, reversal of multidrug resistance, and inhibition of calcium channel [39]. It also induces G1 blockade of the G1 phase of apoptosis and cell cycle in various cell types [40]. In cyclomorusin and site1 of PBP2a interactions, Asn464 and Gln613 were acting as side-chain acceptors and donors, respectively, while the surrounding amino acids were present to provide a hydrophobic environment. Cyclomorusin is a pyranoflavonoid, heterocyclic, and flavonoid lipid molecule, which contains pyran ring fused with a 2-phenyl-1,4-benzopyran backbone. It has been found in the root bark of *Morus alba* (white mulberry) and *Artocarpus altilis* (breadfruit). Its leaves, fruits, roots, and twigs have been used in traditional Chinese medicine [41]. Cyclomorusin plays a crucial role as an inhibitor of platelet aggregation [42].

In the interaction of lipomycin and site1 and site3 of PBP2a, the polar Gln613 and Thr600 and the basic Lys597 formed hydrogen bonds with lipomycin and acted as side-chain donors. The polar Tyr446 and greasy Leu594 acted as backbone donors. Lipomycin is a glycoside and has been reported for its antimicrobial activities, especially against Gram-positive bacteria [43]. In morusin and site1 of the PBP2a interaction, the polar Thr444 acted as a side-chain acceptor. Morusin or mulberrochromene is a flavanoid present in the root bark of *Morus alba* (mulberry) and other Morus species. Morusin has been reported as an antioxidant, anticancer, and antineoplastic agent to suppress cancer cells [44]. Aromadendrin or dihydrokaempferol is a bioactive flavonoid which has been reported from *Pinus sibirica*, *Chioanathus retusus*, and *Afzelia bella* [45]. It has antiviral, antimicrobial, and antidiabetic properties [46]. Aromadendrin has also been reported for its biological activities such as the production of lipopolysaccharide-induced proinflammatory mediators and lipopolysaccharide-induced degradation of I*κ*B [47].

Rosmarinic acid or rosemary is a type of polyphenolic compound that belongs to a class of organic compounds known as coumaric acids. It is present in culinary herbs such as *perilla, rosemary, sage, mint*, and *basil*. These kitchen herbs are used to add flavour to cooking and have several potent physiological effects. Rosmarinic acid contains a cinnamic acid moiety and serves as an antioxidant and antiallergic agent used for the treatment of various allergies, asthma, and lung-associated diseases [48]. Chrysoeriol or scoparol belongs to the class of 3′-o-methylated flavonoids and has been extracted from alfalfa and luteolin. It has anti-inflammatory and antioxidant properties. Chrysoeriol also plays a crucial role as an antineoplastic agent to suppress the proliferation of neoplasm cells [49]. Alpha-lapachone belongs to a group of compounds known as prenyl naphthoquinone lapachol and has been extracted from *Catalpa ovate* [50]. This group of compounds has been reported for multiple biological activities such as antioxidant, anticancer, immunomodulator, antifungal, antibacterial, and anti-inflammatory activities [51].

Phenolic compounds are known for their broad-spectrum antibacterial activity against both Gram-positive and Gram-negative bacterias. They act by disrupting the bacterial cell membrane or inhibiting bacterial enzymes [52]. Phenolic compounds have also been shown to inhibit biofilm formation, which is a common mode of bacterial growth and survival [53]. Phenolic compounds can enhance the activity of antibiotics against bacteria, potentially reducing the required dose and minimizing the development of antibiotic resistance [54]. In asphodoside-D and site1 of PBP2a interaction, the polar Gln613 acted as a side-chain donor, while the polar Gln521 acted as both a side-chain donor and an acceptor. The polar Thr444 acted as both a side-chain and a backbone acceptor. Other polar amino acids which include Thr600 and Tyr446 acted as backbone acceptor and donor, respectively. Asphodoside-D has been reported from *Asphodelus microcarpus* and has antibacterial activities [55]. In the interaction between bacopasaponin A and site1 of PBP2a, the polar Gln613 acted as a side-chain donor, while Gln521 acted as both side-chain donor and acceptor. Polar Thr444 also acted as both side-chain and backbone acceptor, while Thr600 acted as a backbone acceptor. The polar Tyr446 was acting as a backbone donor in this interaction. Bacopasaponin is a constituent of *Bacopa monnieri* and has properties to improve cognition and memory [56].

In diosmin and site1 of PBP2a interaction, polar Ser462 and Ser403 were acting as side-chain and backbone donors, respectively. In this interaction, the polar Asn464 and acidic Glu602 were acting as side-chain donor and acceptor, respectively, while Thr600 acted as a side-chain donor. Diosmin is a flavone and has been found in the plant *Teucrium gnaphalodes*. It has antioxidant and anti-inflammatory properties. It is being used as a drug in several European countries [57]. The interactions between rutin and site1 and site3 of PBP2a showed that polar Thr444 acted as a side-chain acceptor, while acidic Glu447 acted as a side-chain donor. Polar Ser598 was acting as backbone acceptor, and Tyr446 was having strong receptor contact with rutin. Rutin is a flavonol glycoside and has been found in many plants including *buckwheat, viola, forsythia*, and *hydrangea*. It has been used therapeutically to improve capillary health. It also has antiallergic, anti-inflammatory, and anticancer properties [58].

Among the main proteins of *S. aureus*, PBP2a is one of the proteins that has caused resistance against *β*-lactam antibiotics. A lateral binding site, which is known as an allosteric site, can be used to modulate the active site of PBP2a. In this way, the *β*-lactam antibiotics which were inactivated previously can now reach and inactivate PBP2a and hence inhibit the growth of the superbug MRSA. In the current study, we have, therefore also targeted the allosteric site of PBP2a because the binding of the ligand molecule with the allosteric site triggers a series of conformational changes in the entrie protein, leading to easy access and inhibition of the active site of PBP2a by *β*-lactam antibiotics or similar drugs. In this study, all selected 9 phytochemcials revealed strong binding interactions with the allosteric site of PBP2a except tetrandrine and therefore could be used to target the allosteric site for the modulation of the active site of the protein. In a study, Ibrahim et al. [59] also explored allosteric inhibitors of PBP2a using molecular docking study. On the basis of the binding affinities of ligands with the receptor, the compounds eMol26313223 and eMol26314565 were found to be strong allosteric inhibitors of PBP2a. Ceftaroline (i.e., an MRSA-active antibiotic) is the first example of a *β*-lactam antibiotic, which was used to mediate an action to induce the opening of the active site after binding to the allosteric site of the PBP2a [60].

Antibiotics have potential side effects such as cytotoxicity and nephrotoxicity. These antibiotics have also been reported to have adverse GI effects, including nausea, vomiting, diarrhea, increased salivation, stomatitis, weight loss, decreased appetite, and anorexia [61]. Till now, many drugs have been found effective against bacterial resistance, but the trend of bacterial gene modifications increases the risk of resistance and the prevalence of infection. PBP2a is an antibiotic-resistant transpeptidase that needs suitable and potent inhibitors to prevent bacterial production.

## 5. Conclusion

The present study was based on the molecular docking and site finding in penicillin-binding protein-2a (PBP2a) of MRSA. A total of 284 ligands were analyzed for their interactions with the active and allosteric sites of the receptor protein. Nine ligands (i.e., cyanidin, tetrandrine, cyclomorusin, lipomycin, morusin, aromadendrin, rosmarinic acid, chrysoeriol, and *α*-lapachoneon) the basis of their S-scores, binding interactions, and drug-likeness were selected as active antagonists of PBP2a. The results of the study indicate that the selected ligands could be used as potent antagonists of PBP2a of MRSA. Further *in vivo* studies are required to verify the findings.

## Figures and Tables

**Table 1:** Physiochemical properties of PBP2a.

S. No.	Property	Value
1	Sequence length	642
2	Molecular weight	73309.89
3	Charge	7.5
4	Isoelectric point	8.07
5	A_280_ molar extinction coefficients Abs 0.1% (=1 g/l)	90650 M^- 1^ cm^- 1^ 1.237
6	The improbability of expression in inclusion bodies	0.780
7	Aliphatic index	78.97
8	Molecular formula	C_3251_H_5157_N_871_O_1022_S_16_
9	Total number of atoms	10317
10	Grand average of hydropathicity (GRAVY)	-0.802
11	Instability index	31.30
12	The estimated half-life is	1 hour (mammalian reticulocytes, in vitro). 30 min (yeast, in vivo). >10 hours (*Escherichia coli*, *in vivo*).

**Table 2:** Identified active sites and an allosteric site of PBP2a by MOE software.

Site	Site no.	Size	PLB	Hyd	Residues
Active sites	1	137	3.42	23	SER403, LYS430, THR444, ARG445, TYR446, GLU447, SER461, SER462, ASP463, ASN464, TYR519, GLN521, SER598, GLY599, THR600, ALA601, GLU602, GLY611, GLN613, GLY615, TRP616, ASP635, LYS639, GLY640, MET641, ALA642, SER643, TYR644, ASN645
	2	54	1.70	27	TYR344, GLY345, SER346, GLU389, LEU392, LYS394, ILE397, THR398, THR399, SER400, LEU525, PRO528, GLU602, LEU603, LYS604, MET605, ILE614, ASN632, VAL633, LYS634
	3	40	1.23	24	MET575, GLN576, GLN577, VAL579, ILE587, TYR588, LEU5594, ILE595, GLY596, LYS597, SER598, PHE617, SER643, ALA646
	4	27	0.80	12	ASP343, TYR344, LYS388, VAL633, LYS634, ASP635, VAL636, GLN637, ASP638, LYS639, MET641, TYR644
	5	40	0.76	13	TYR441, ASN442, THR444, ASP16, TYR519, GLN521, GLU602, LEU602, LEU603, LYS604, MET605, LYS606, THR610, GLY611, ARG612
	6	8	-0.34	12	LYS426, ILE427, ASP428, TRP432, ILE465, ARG469
	7	16	-0.35	5	TYR344, LYS387, GLU389, ARG612, LYS634, ASP635, VAL636
	8	16	-0.38	9	LEU414, LYS417, LEU419, ASP420, ASP420, ASP421, ASN567, LEU570
	9	7	-1.08	4	SER504, ASN505, LYS506, ASN507
	10	6	-1.15	5	SER376, GLU378, GLU379, LYS382

Allosteric site	2	65	1.58	24	ASN146, LYS148, SER149, GLY271, TYR272, LYS273, ASP275, ALA276, VAL277, GLN292, HIS293, GLU294, ASP295, GLY296, TYR297, LYS316, LYS319, ASP320, GLY321

PLB: propensity for ligand binding score; Hyd: number of hydrophobic contact atoms; Residues: residues that make up the active site; Size: number of alpha spheres comprising the site.

**Table 3:** ADME properties of phytochemicals that followed Ro5 rule.

Sr. no.	Names	MW (g/mol)	RB	HBA	HBD	TPSA (Å^2^)	Consensus log *P*_o/w_	GI absorption	Violations	S-score (kcal/mol)	Lipinski ‘s test
1	Cyanidin	287.24	1	6	5	114.3	0.56	High	0	-16.061	Passed
2	Tetrandrine	622.75	4	8	0	61.86	5.46	High	1	-14.093	Passed
3	Cyclomorusin	418.44	1	6	2	89.13	4.22	High	0	-13.896	Passed
4	Lipomycin	587.7	13	9	4	153.8	3.4	Low	1	-13.761	Passed
5	Morusin	420.45	3	6	3	100.1	4.35	High	0	-13.755	Passed
6	Aromadendrin	288.25	1	6	4	107.2	1.02	High	0	-13.501	Passed
7	Rosmarinic acid	360.31	7	8	5	144.5	1.58	Low	0	-12.715	Passed
8	Chrysoeriol	300.26	2	6	3	100.1	2.18	High	0	-12.65	Passed
9	*α*-Lapachone	258.27	0	4	1	63.6	2.34	High	0	-12.437	Passed

MW:molecular weight; RB: number of rotatable hydrogen bonds; HBA: number of hydrogen bond accepter; HBD: number of hydrogen bond donor; TPSA: topological polar surface area, Logp: lippophilicity coefficient; GI absorption: gastrointestinal absorption.

**Table 4:** Top 9 phytochemicals that follow Ro5.

Sr. no.	Names and PubChem CID	2D structure	Active site			Allosteric site		
			S-score (kcal/mol)	RMSD	Site	S-score (kcal/mol)	RMSD	Site
1	Cyanidin 128861	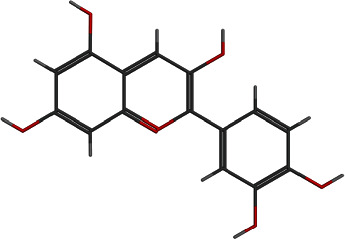	-16.061	1.42	3	-13.199	1.78	2
2	Tetrandrine 73078	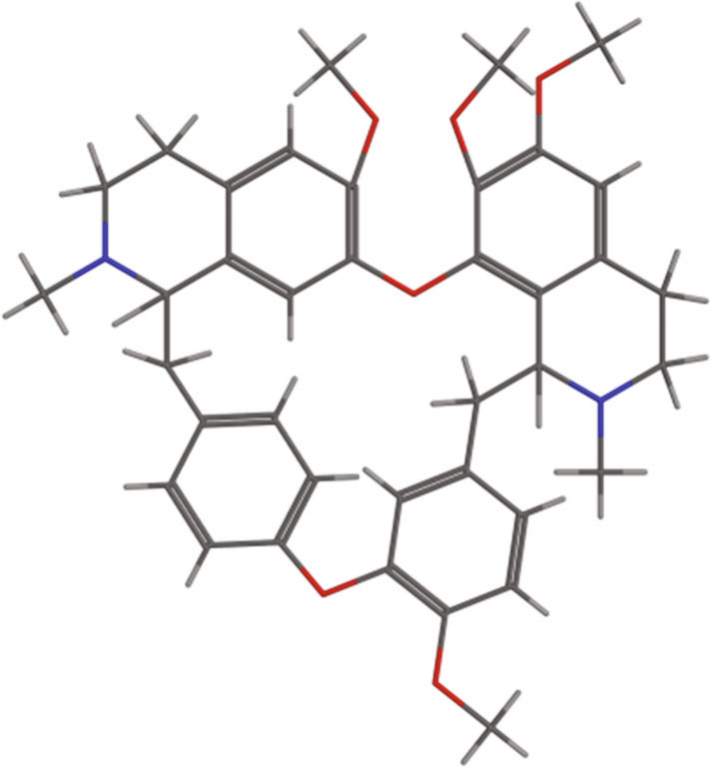	-14.093	1.40	1	-6.634	2.70	2
3	Cyclomorusin 5481969	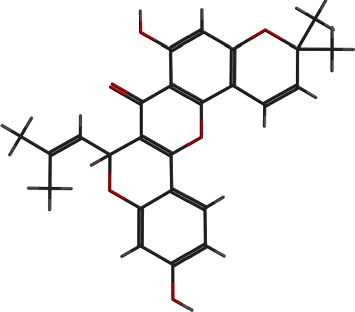	-13.896	1.87	1	-12.013	1.71	2
4	Lipomycin 54723870	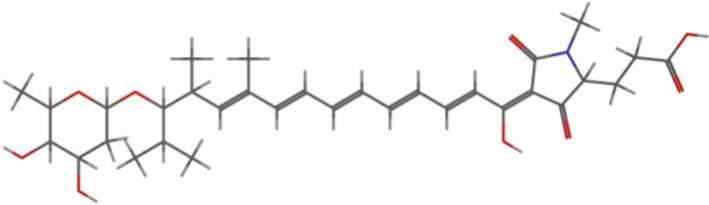	-13.761	2.70	1, 3	-12.819	2.13	2
5	Morusin 5281671	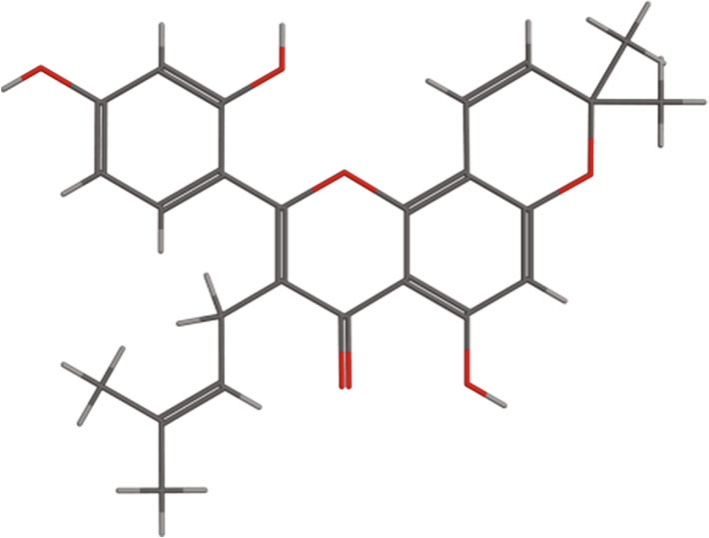	-13.755	1.03	1	-14.843	2.01	2
6	Aromadendrin 122850	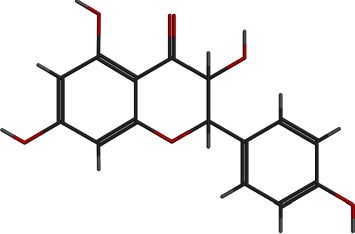	-13.501	1.27	1	-10.738	0.71	2
7	Rosmarinic acid 5281792	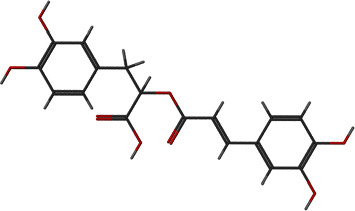	-12.715	1.73	1, 3	-10.649	2.34	2
8	Chrysoeriol 5280666	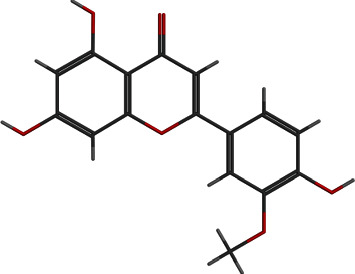	-12.65	0.46	3	-9.874	2.08	2
9	*α*–Lapachone 5320006	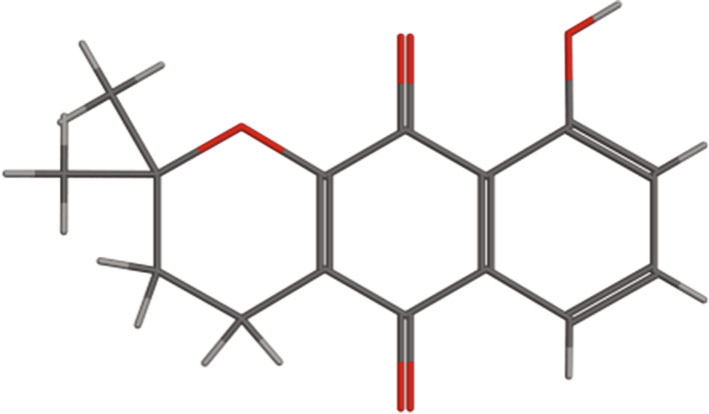	-12.437	0.65	3	-10.520	1.81	2

**Table 5:** Toxicities and bioactivities of phytochemicals that followed Ro5.

Names	Toxicities predicted by DataWarrior software				Bioactivities predicted by Molinspiration database					
	Mutagenic	Tumorigenic	Reproductive effective	Irritant	GPCR ligand	Ion channel modulator	Kinase inhibitor	Nuclear receptor ligand	Protease inhibitor	Enzyme inhibitor
Cyanidin	None	None	None	None	-0.13	-0.09	0.02	0.09	-0.3	0.01
Tetrandrine	None	None	None	None	-0.11	-0.77	-0.62	-0.71	-0.13	-0.44
Cyclomorusin	None	None	None	None	0.1	-0.26	0.01	0.68	-0.12	0.42
Lipomycin	None	None	None	None	0.23	-0.22	-0.29	-0.17	0.31	0.47
Morusin	None	None	None	None	0.11	-0.2	0.03	0.77	-0.12	0.47
Aromadendrin	None	None	None	None	0.05	0.03	-0.07	0.26	0.03	0.29
Rosmarinic acid	None	None	None	None	0.17	-0.08	-0.18	0.57	0.15	0.24
Chrysoeriol	None	None	None	None	-0.05	-0.14	0.25	0.32	-0.26	0.21
*α*-Lapachone	None	None	None	None	-0.16	-0.05	-0.12	0.41	-0.03	0.44

Mutagenic: cause damage in genetic material; Tumorigenic: tumor causing; Reproductive Effective: interfere in normal reproduction; Irritant: causes slight inflammation or other discomforts to the body; if bioactivity score of a phytochemical is > 0 then it is active; if (-5.0 - 0.0) then moderately active; if < -5.0 then it is inactive.

## Data Availability

The data used to support the findings of this study are available from the corresponding author upon request.

## Abbreviations

MRSA: Methicillin-resistant staphylococcus aureus
SCCmec: Staphylococcal chromosome cassette mec
PBP2a: Penicillin-binding protein 2a
NAG: N-acetylglucosamine
NAM: N-acetylmuramic acid.

## Supplementary Materials

Supplementary Materials Figure S1: 2D and 3D interactions of asphoside-D with site1 of PBP2a. Figure S2: 2D and 3D interactions of bacopasaponin A with site1 of PBP2a. Figure S3: 2D and 3D interactions of diosmin with site1 of PBP2a. Figure S4: 2D and 3D interactions of rutin with site1 of PBP2a. Figure S5: 2D and 3D interactions of ticarcillin with site1 of PBP2a. Figure S6: 2D and 3D interactions of methicillin with site1 of PBP2a. Figure S7: 2D and 3D interactions of penicillin-V with site1 of PBP2a. Figure S8: 2D and 3D interactions of piperacillin with site1 and site3 of PBP2a. Figure S9: 2D and 3D interactions of carbenicillin with site1 of PBP2a. Figure S10: 2D and 3D interactions of amikacin with site1 of PBP2a. Figure S11: 2D and 3D interactions of gentamicin with site1 of PBP2a. Figure S12: 2D and 3D interactions of vancomycin with site1 and site5 of PBP2a. Figure S13: 2D and 3D interactions of oxytetracycline with site1 of PBP2a. Figure S14: 2D and 3D interactions of cefoxitin with site1 of PBP2a. Figure S15: 2D and 3D interactions of N-acetyl-muramic acid with site1 of PBP2a. Figure S16: 2D and 3D interactions of cyanidin with the PBP2a allosteric site. Figure S17: 2D and 3D interactions of tetrandrine with PBP2a allosteric site. Figure S18: 2D and 3D interactions of cyclomorusin with the PBP2a allosteric site. Figure S19: 2D and 3D interactions of lipomycin with the PBP2a allosteric site. Figure S20: 2D and 3D interactions of morusin with the PBP2a allosteric site. Figure S21: 2D and 3D interactions of aromadendrin with the PBP2a allosteric site. Figure S22: 2D and 3D interactions of rosmarinic acid with the PBP2a allosteric site. Figure S23: 2D and 3D interactions of chrysoeriol with the PBP2a allosteric site. Figure S24: 2D and 3D interactions of *α*-lapachone with the PBP2a allosteric site. Table S1: phytochemicals that do not follow the Ro5. Table S2: ADME properties of phytochemicals that do not follow Ro5. Table S3: toxicities and bioactivities of phytochemicals that do not follow Ro5. Table S4: name, PubChem ID, 2D structure, binding affinities and binding site of penicillins. Table S5: ADME properties of penicillins. Table S6: toxicities of penicillins predicted by DataWarrior Software. Table S7: name, PubChem ID, 2D structure, score of general antibiotics, and binding site of PBP2a. Table S8: drug-likeness properties of general antibiotics. Table S9: toxicities of general antibiotics predicted by DataWarrior software.
