# Supplementary material for: In Silico Method for the Screening of Phytochemicals against Methicillin-Resistant Staphylococcus Aureus
Source: Biomed Res Int. 2023 May 18;2023:5100400. doi: 10.1155/2023/5100400 (PMC10212682; doi:10.1155/2023/5100400)
Supplement: Supplementary Materials — Figure S1: 2D and 3D interactions of asphoside-D with site1 of PBP2a. Figure S2: 2D and 3D interactions of bacopasaponin A with site1 of PBP2a. Figure S3: 2D and 3D interactions of diosmin with site1 of PBP2a. Figure S4: 2D and 3D interactions of rutin with site1 of PBP2a. Figure S5: 2D and 3D interactions of ticarcillin with site1 of PBP2a. Figure S6: 2D and 3D interactions of methicillin with site1 of PBP2a. Figure S7: 2D and 3D interactions of penicillin-V with site1 of PBP2a. Figure S8: 2D and 3D interactions of piperacillin with site1 and site3 of PBP2a. Figure S9: 2D and 3D interactions of carbenicillin with site1 of PBP2a. Figure S10: 2D and 3D interactions of amikacin with site1 of PBP2a. Figure S11: 2D and 3D interactions of gentamicin with site1 of PBP2a. Figure S12: 2D and 3D interactions of vancomycin with site1 and site5 of PBP2a. Figure S13: 2D and 3D interactions of oxytetracycline with site1 of PBP2a. Figure S14: 2D and 3D interactions of cefoxitin with site1 of PBP2a. Figure S15: 2D and 3D interactions of N-acetyl-muramic acid with site1 of PBP2a. Figure S16: 2D and 3D interactions of cyanidin with the PBP2a allosteric site. Figure S17: 2D and 3D interactions of tetrandrine with PBP2a allosteric site. Figure S18: 2D and 3D interactions of cyclomorusin with the PBP2a allosteric site. Figure S19: 2D and 3D interactions of lipomycin with the PBP2a allosteric site. Figure S20: 2D and 3D interactions of morusin with the PBP2a allosteric site. Figure S21: 2D and 3D interactions of aromadendrin with the PBP2a allosteric site. Figure S22: 2D and 3D interactions of rosmarinic acid with the PBP2a allosteric site. Figure S23: 2D and 3D interactions of chrysoeriol with the PBP2a allosteric site. Figure S24: 2D and 3D interactions of α-lapachone with the PBP2a allosteric site. Table S1: phytochemicals that do not follow the Ro5. Table S2: ADME properties of phytochemicals that do not follow Ro5. Table S3: toxicities and bioactivities of phytochemicals that do no [file 5100400.f1.docx]

***In silico* method for the screening of phytochemicals against methicillin-resistant *Staphylococcus aureus***

Riaz Tabassum^1†^, Sumaira Kousar^2†^, Ghulam Mustafa^3*^, Amer Jamil^1^ and Syed Awais Attique^4^

^1^Department of Biochemistry, University of Agriculture, Faisalabad-38040, Pakistan

^2^Department of Biochemistry, Government College Women University, Faisalabad, Pakistan

^3^Department of Biochemistry, Government College University, Faisalabad-38000, Pakistan

^4^School of Interdisciplinary Engineering & Science (SINES), National University of Sciences & Technology (NUST), Islamabad, Pakistan

^*^Corresponding author’s E-mail: gmustafa_uaf@yahoo.com

^†^These two authors contributed equally

***Supplementary Materials***


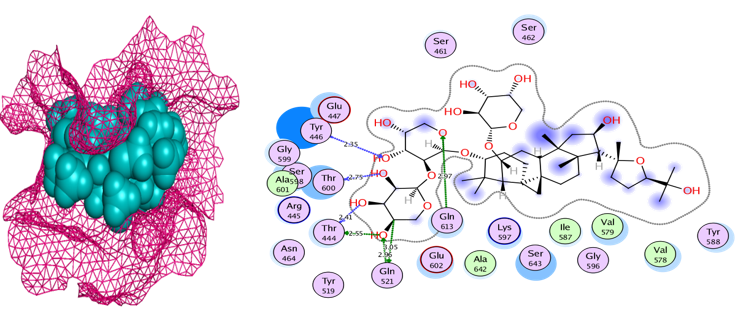


**Figure S1: 2D and 3D interactions of asphoside-D** **with site1 of PBP2a**


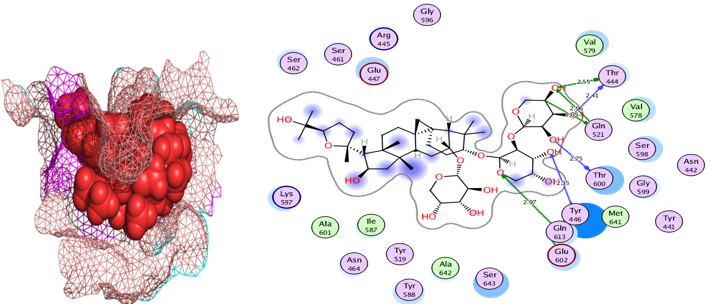


**Figure S2: 2D and 3D interactions of bacopasaponin A** **with site1 of PBP2a**


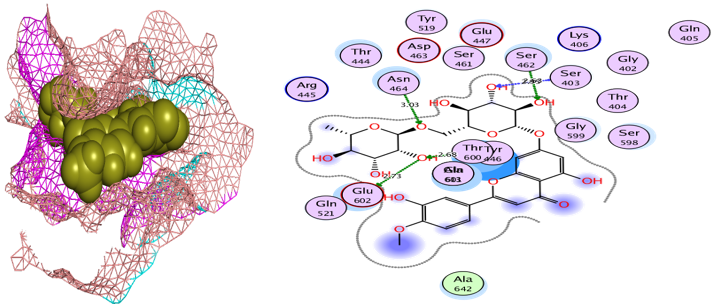


**Figure S3: 2D and 3D interactions of diosmin with site1 of PBP2a**


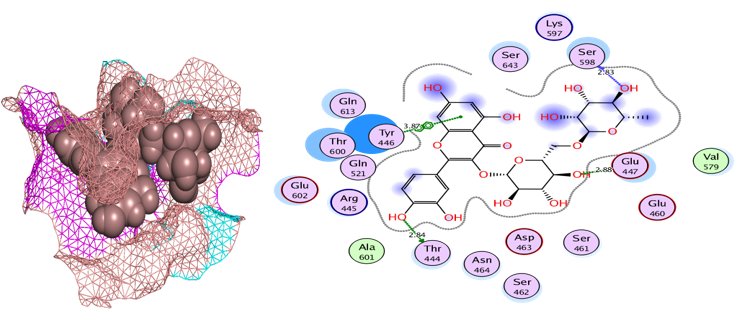


**Figure S4: 2D and 3D interactions of rutin with site1 of PBP2a**


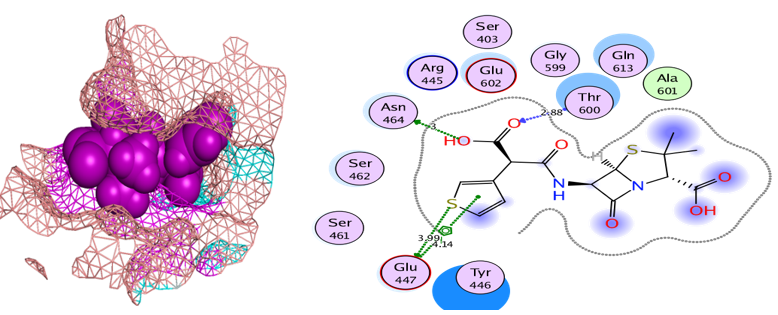


**Figure S5: 2D and 3D interactions of ticarcillin with site1 of PBP2a**


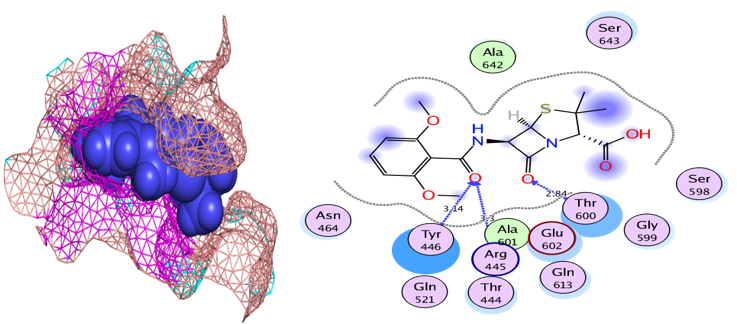


**Figure S6: 2D and 3D interactions of methicillin with site1 of PBP2a**


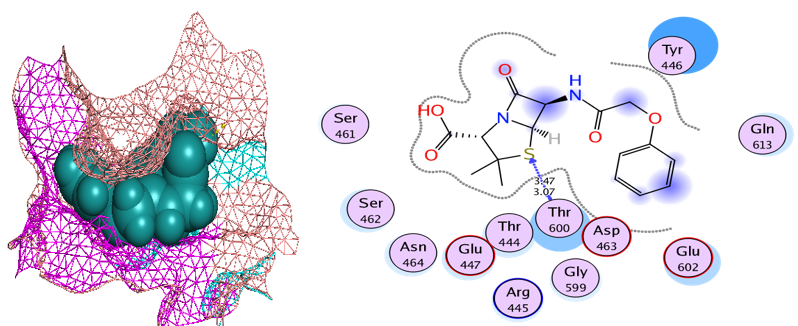


**Figure S7: 2D and 3D interactions of penicillin-V** **with site1 of PBP2a**


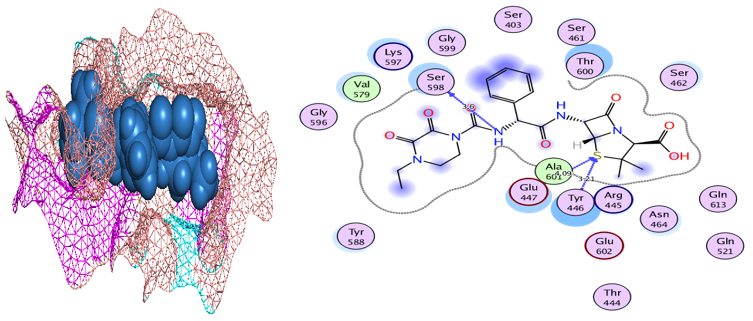


**Figure S8: 2D and 3D interactions of piperacillin** **with site1 and site3 of PBP2a**


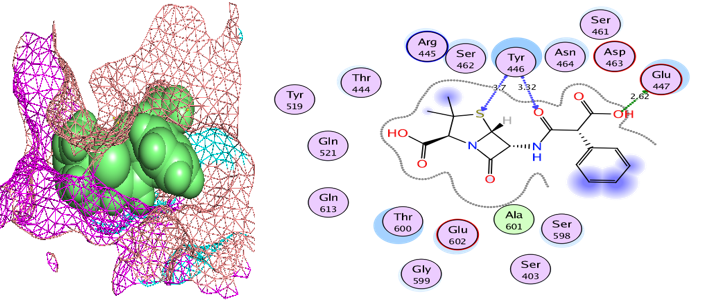


**Figure S9: 2D and 3D interactions of carbenicillin** **with site1 of PBP2a**


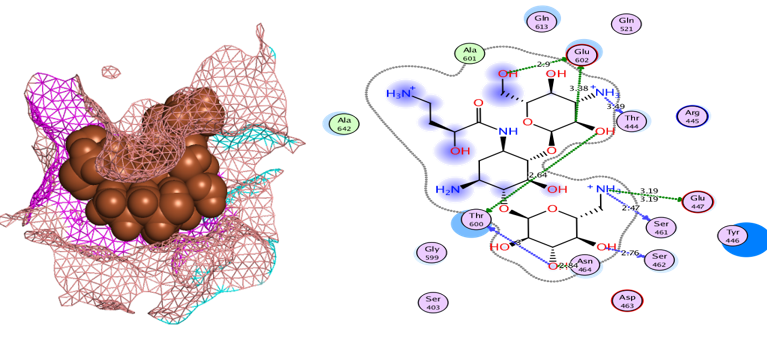


**Figure S10: 2D and 3D interactions of amikacin with site1 of PBP2a**


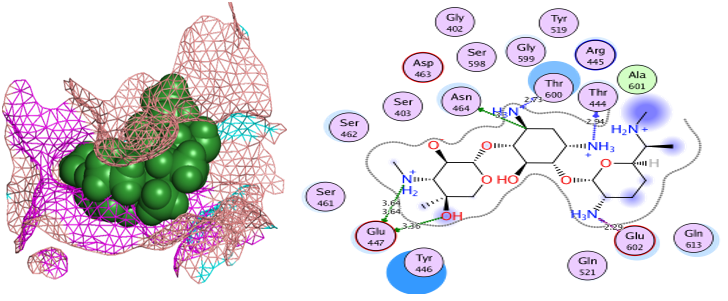


**Figure S11: 2D and 3D interactions of gentamicin with site1 of PBP2a**


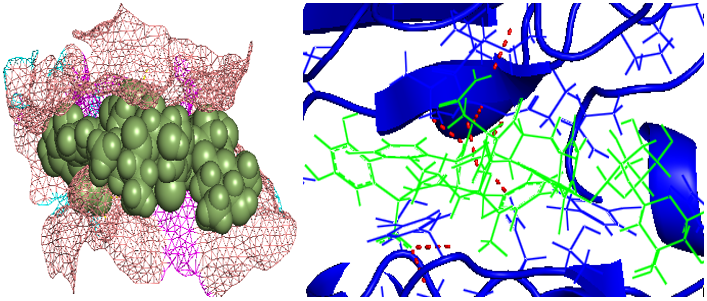


**Figure S12: 2D and 3D interactions of vancomycin with site1 and site5 of PBP2a**


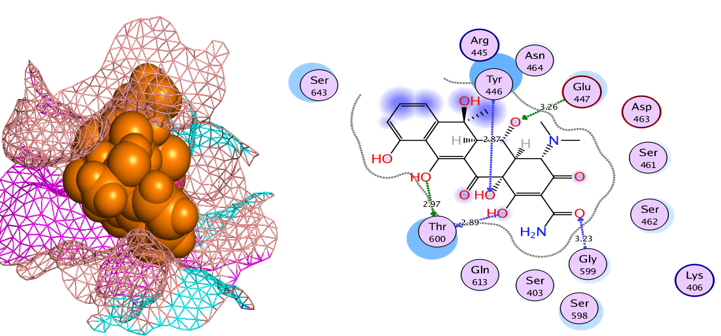


**Figure S13: 2D and 3D interactions of oxytetracycline with site1 of PBP2a**


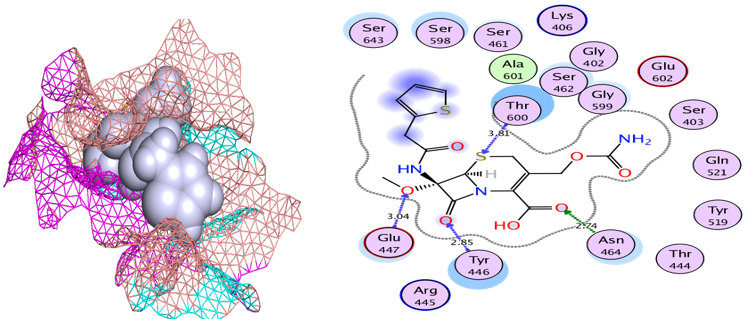


**Figure S14: 2D and 3D interactions of cefoxitin** **with site1 of PBP2a**

| **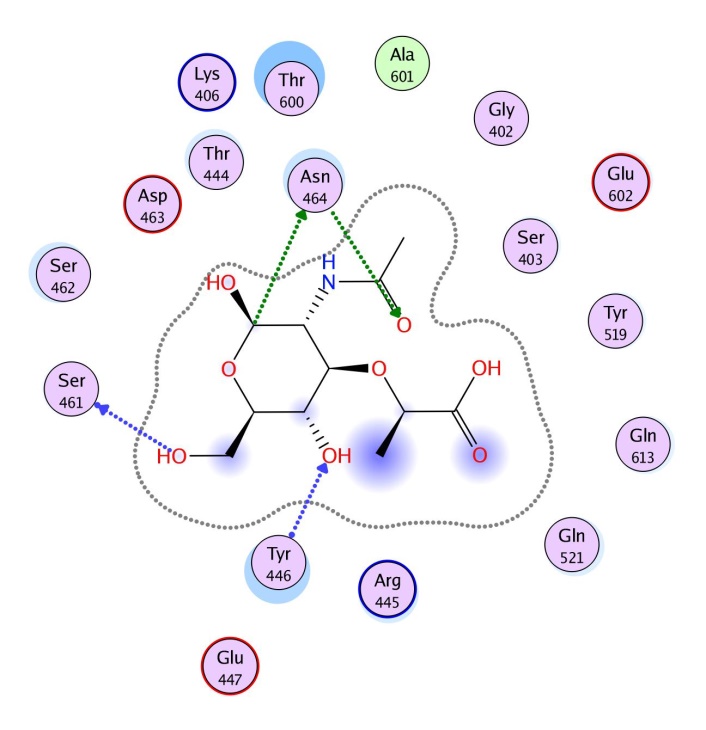** | **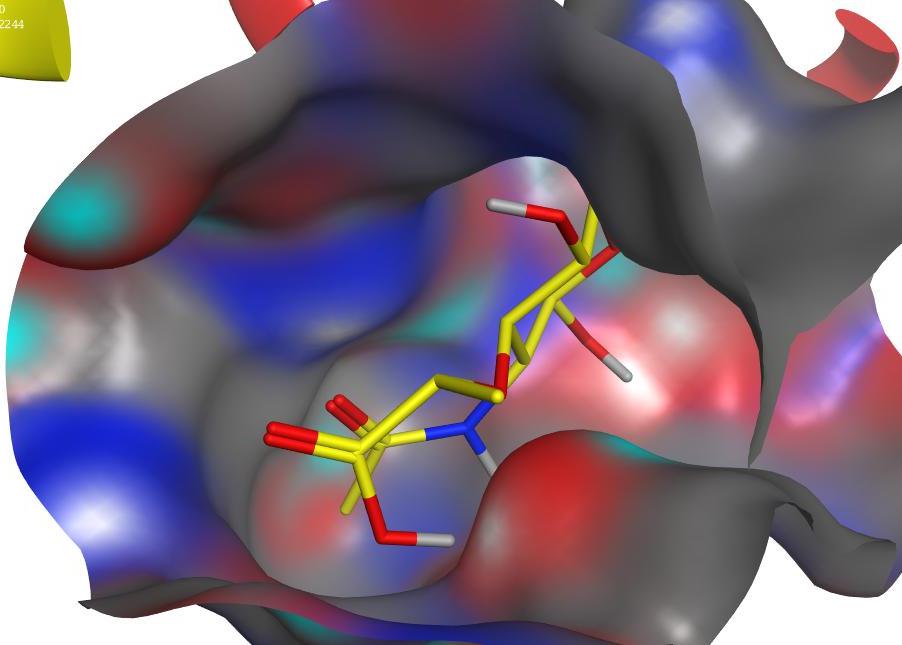** |
| --- | --- |

**Figure S15: 2D and 3D interactions of N-acetyl-muramic acid with site1 of PBP2a**

| **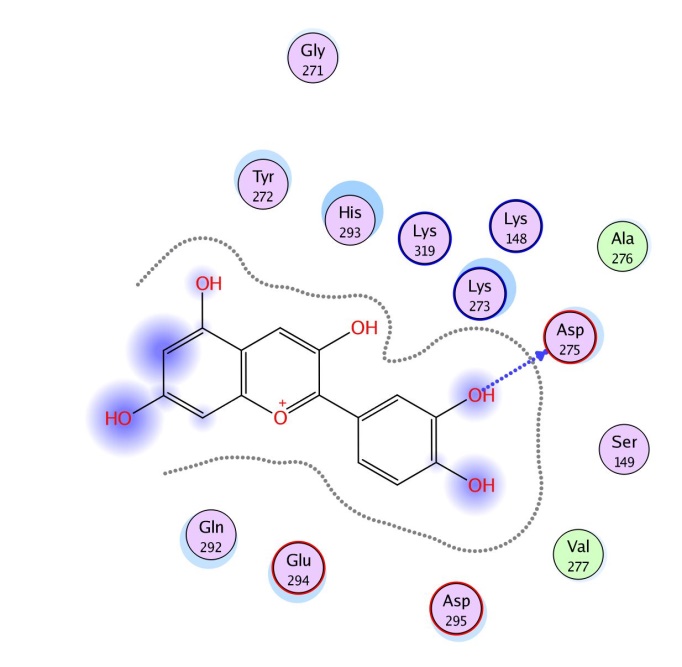** | **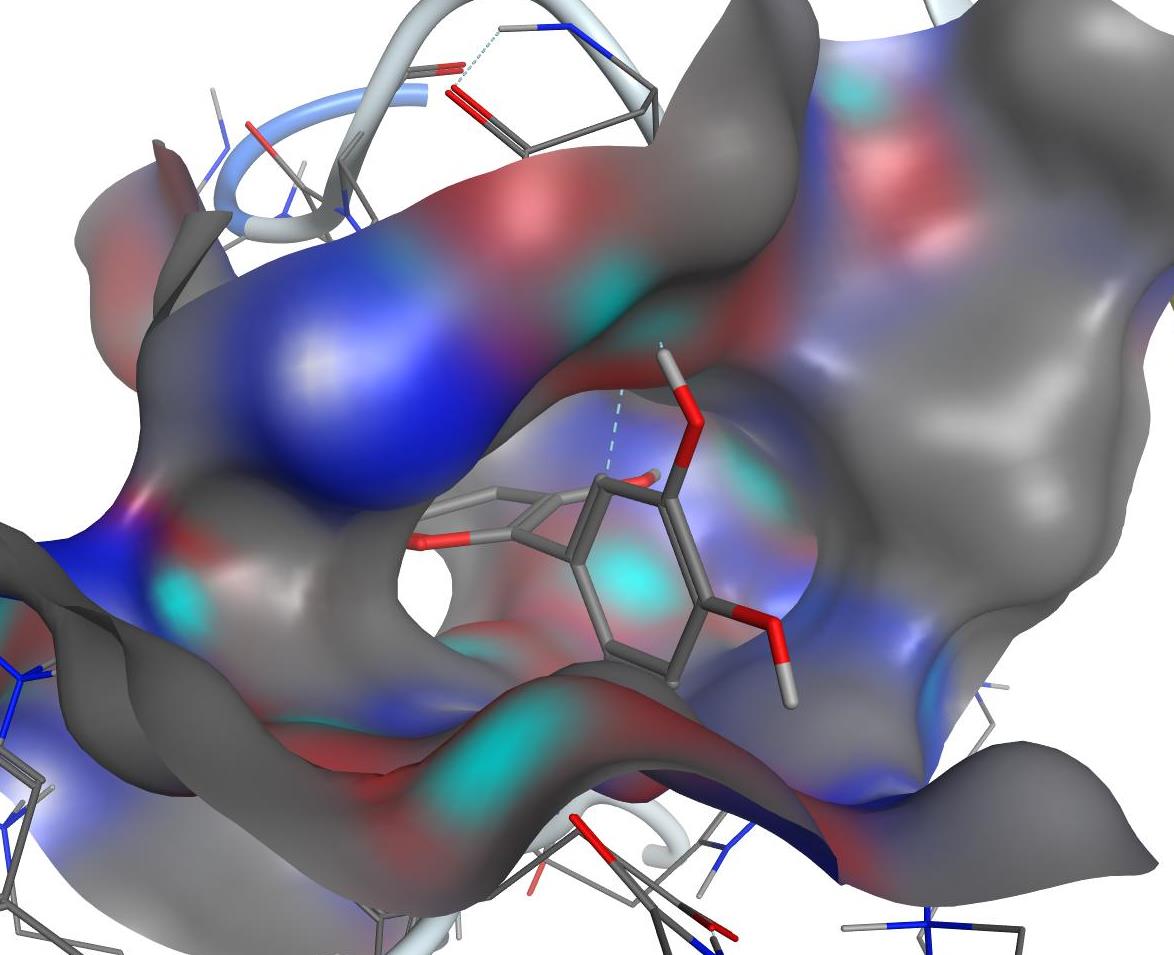** |
| --- | --- |

**Figure S16: 2D and 3D interactions of cyanidin with PBP2a allosteric site**

| **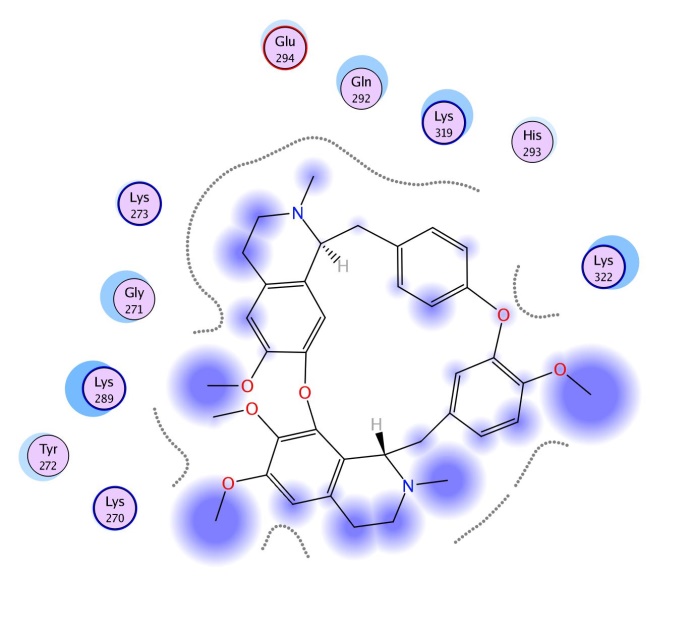** | **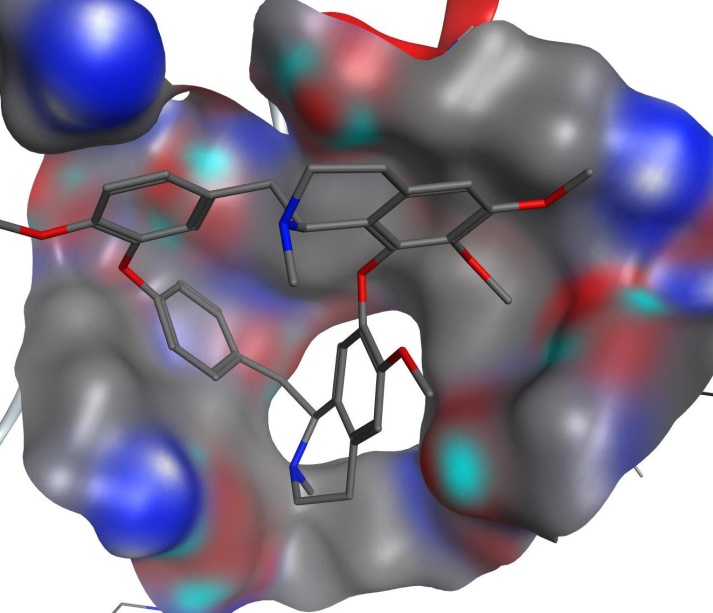** |
| --- | --- |

**Figure S17: 2D and 3D interactions of tetrandrine with PBP2a allosteric site**

| **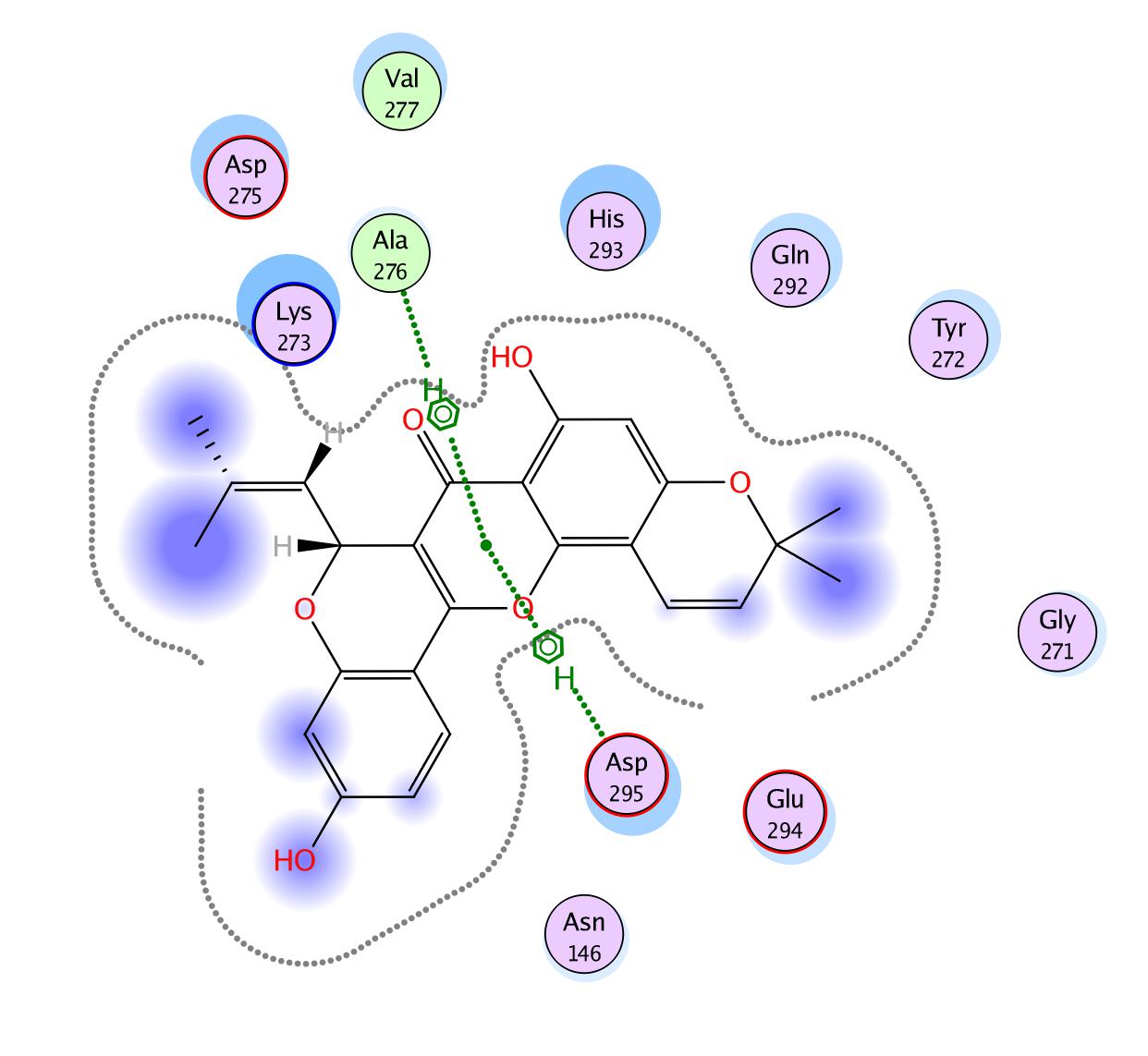** | **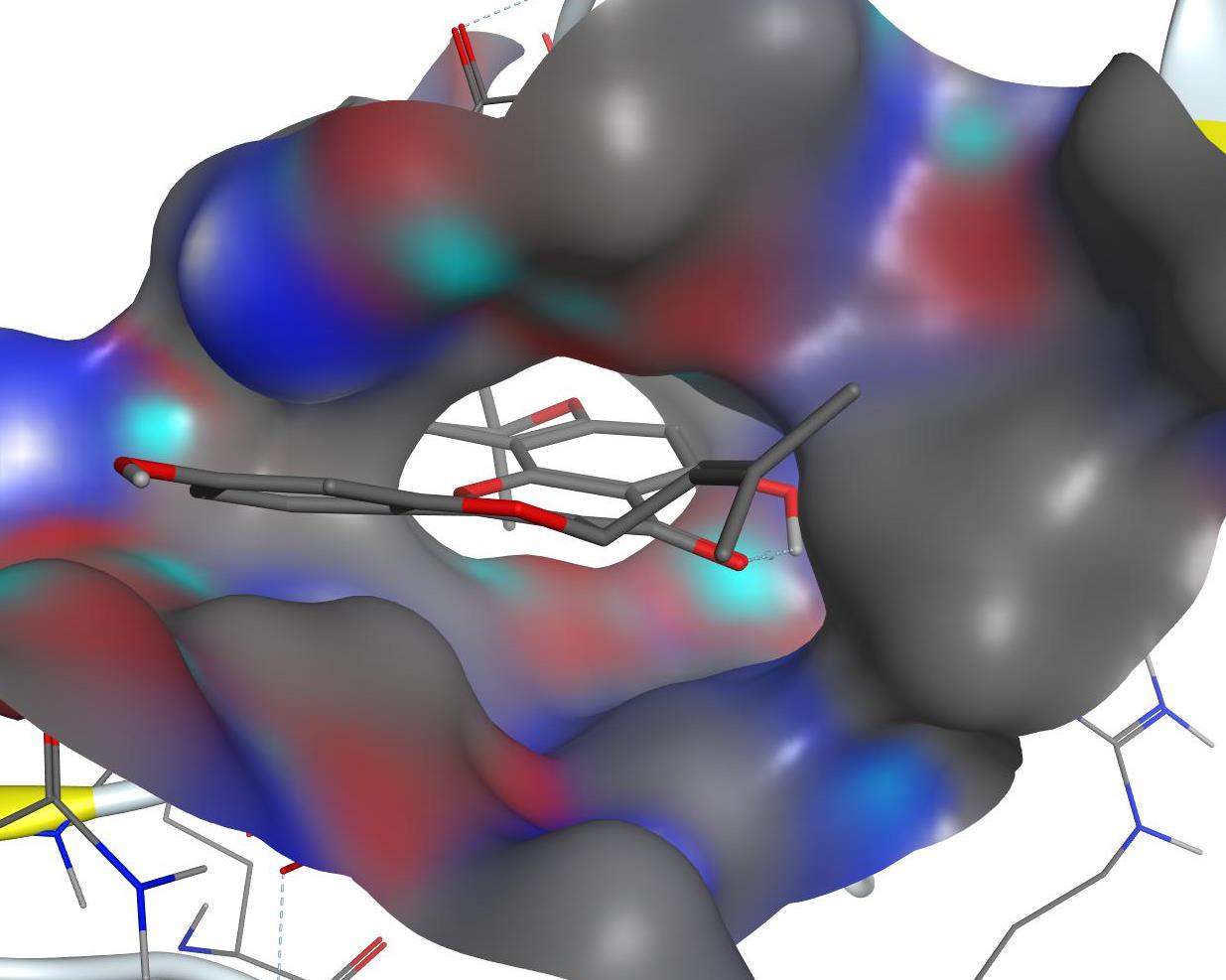** |
| --- | --- |

**Figure S18: 2D and 3D interactions of cyclomorusin with PBP2a allosteric site**

| **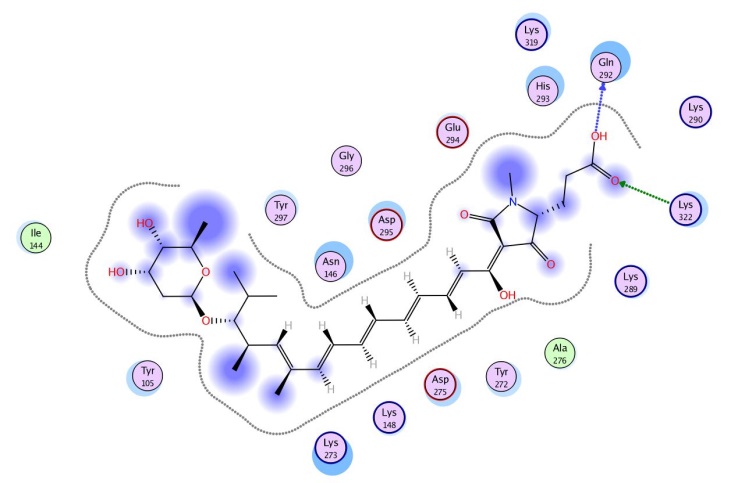** | **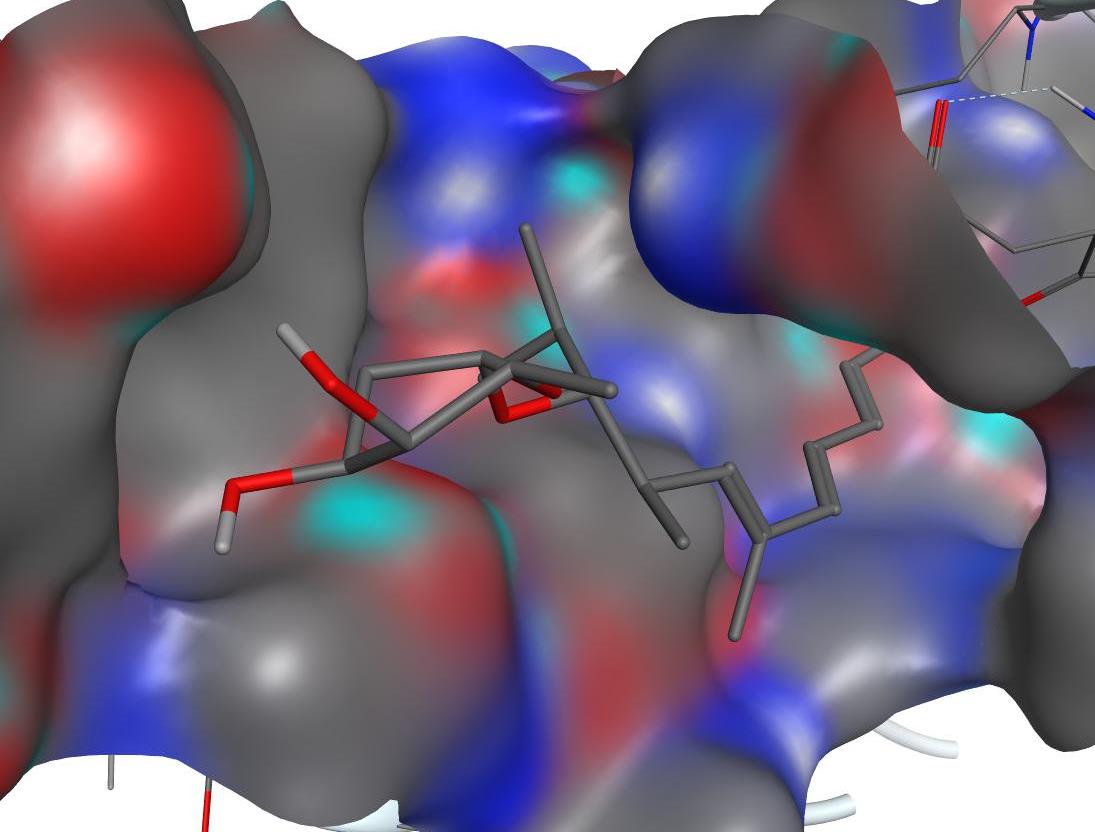** |
| --- | --- |

**Figure S19: 2D and 3D interactions of lipomycin with PBP2a allosteric site**

| **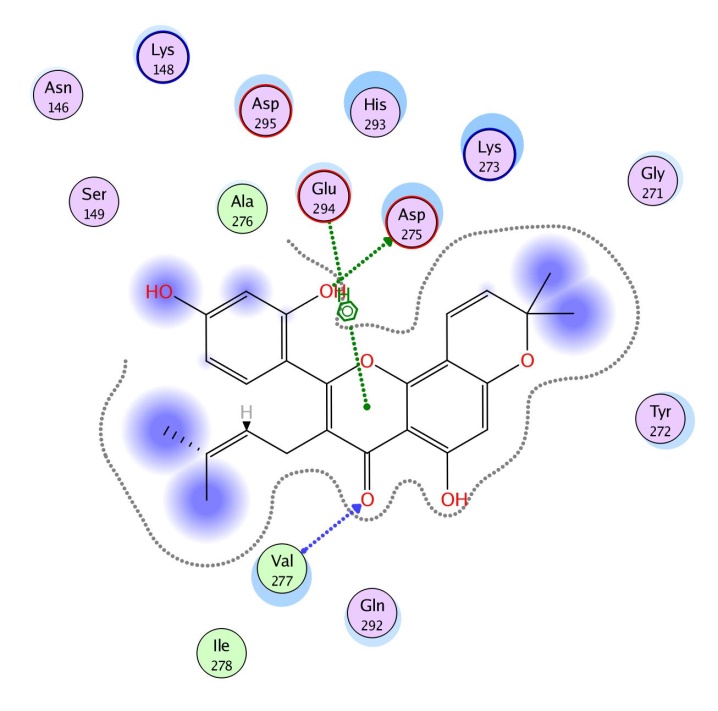** | **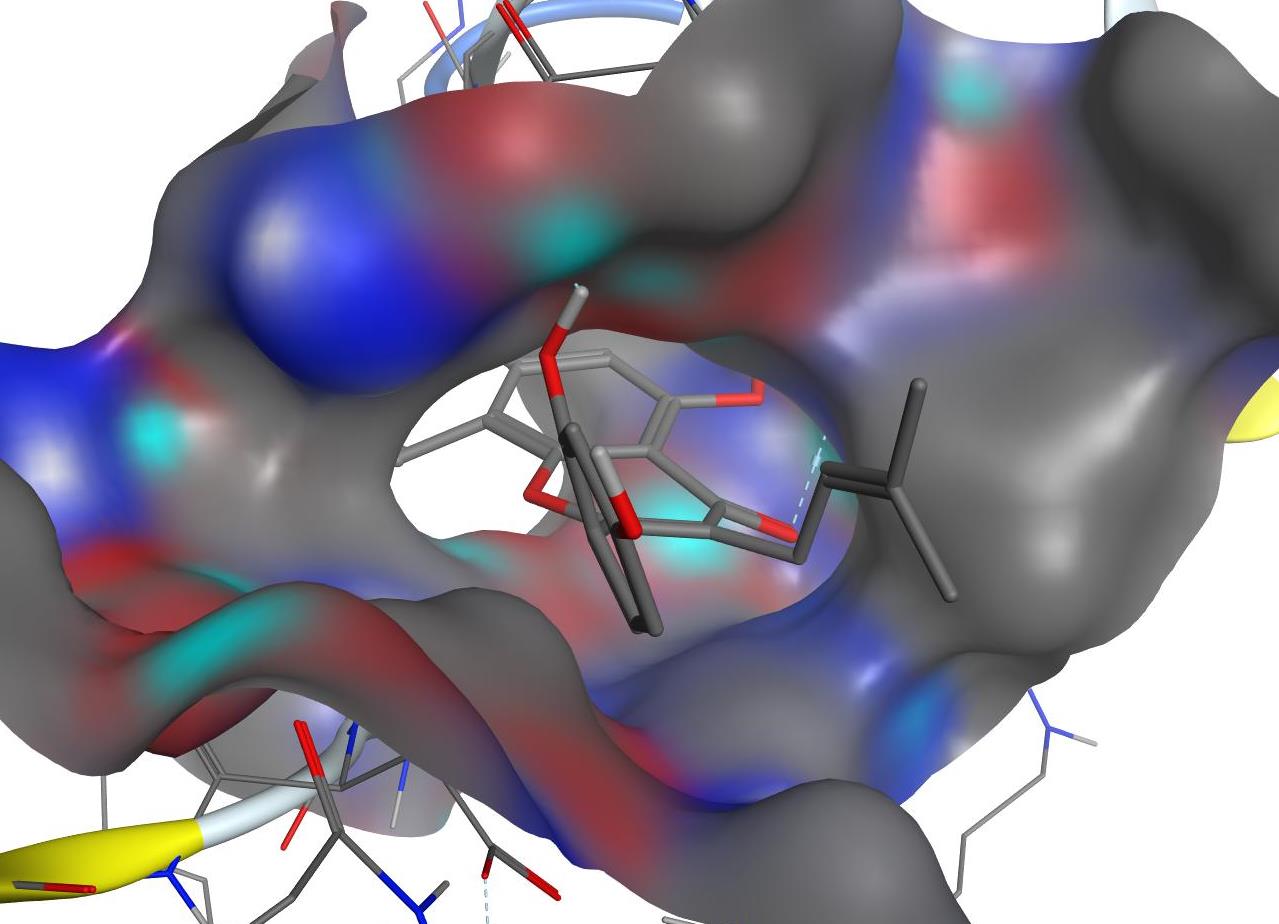** |
| --- | --- |

**Figure S20: 2D and 3D interactions of morusin with PBP2a allosteric site**

| **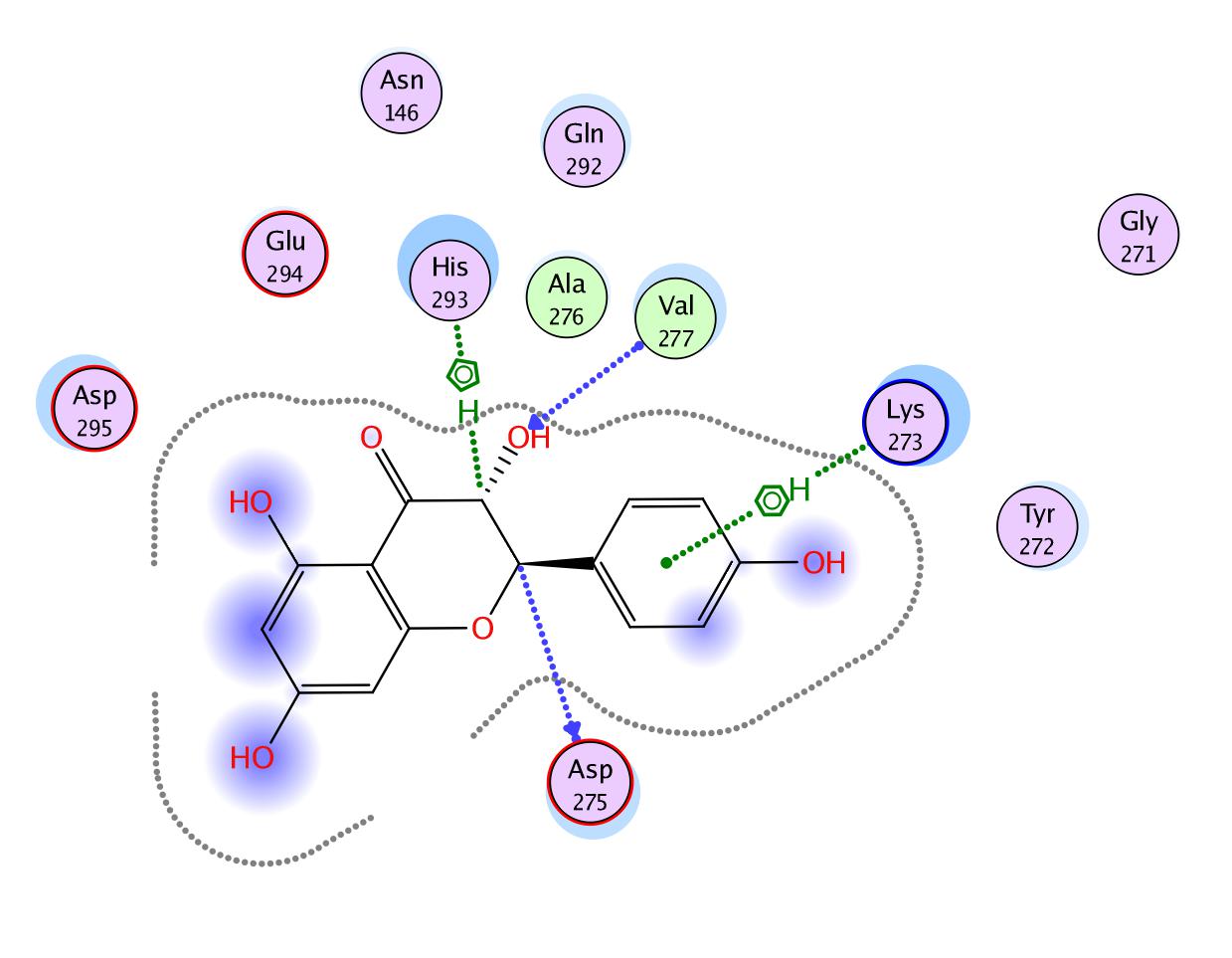** | **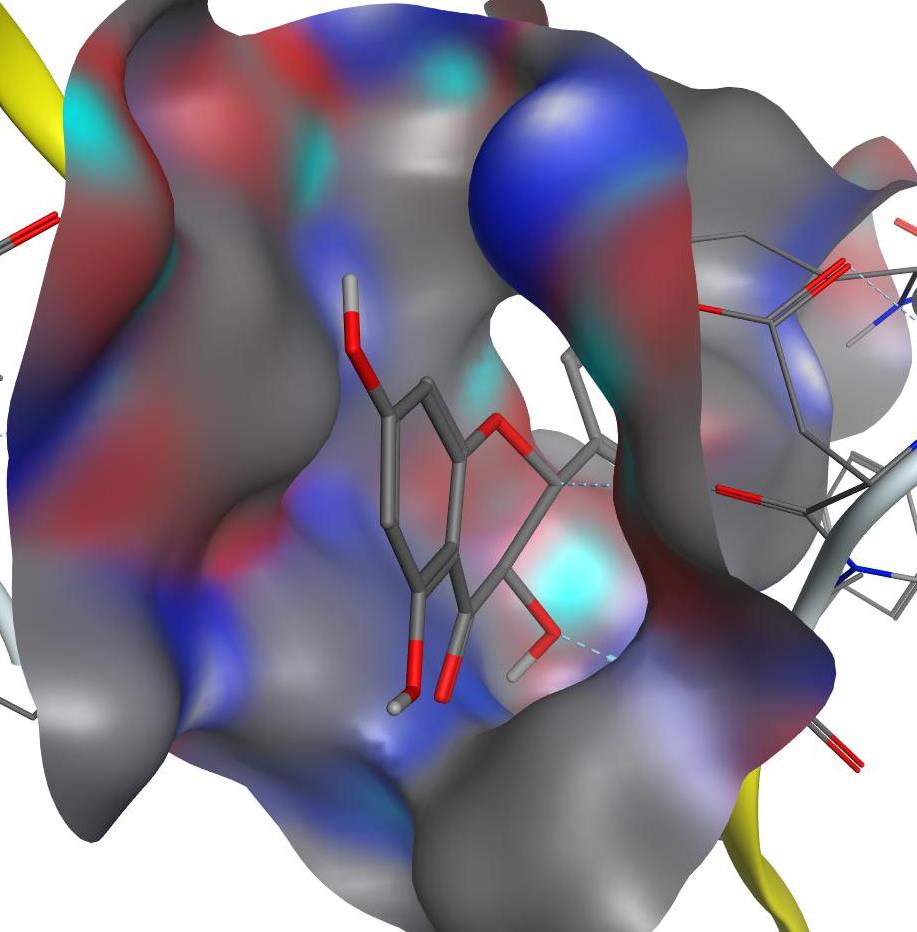** |
| --- | --- |

**Figure S21: 2D and 3D interactions of aromadendrin with PBP2a allosteric site**

| **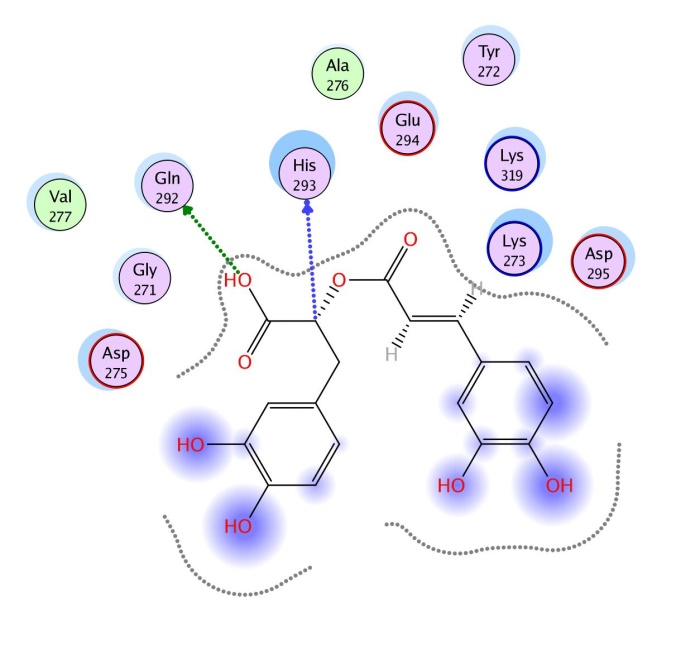** | **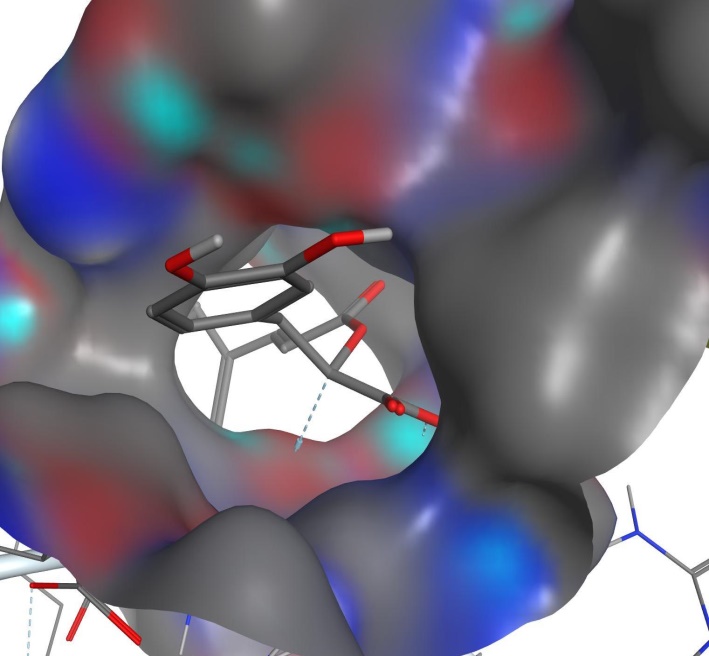** |
| --- | --- |

**Figure S22: 2D and 3D interactions of rosmarinic acid with PBP2a allosteric site**

| **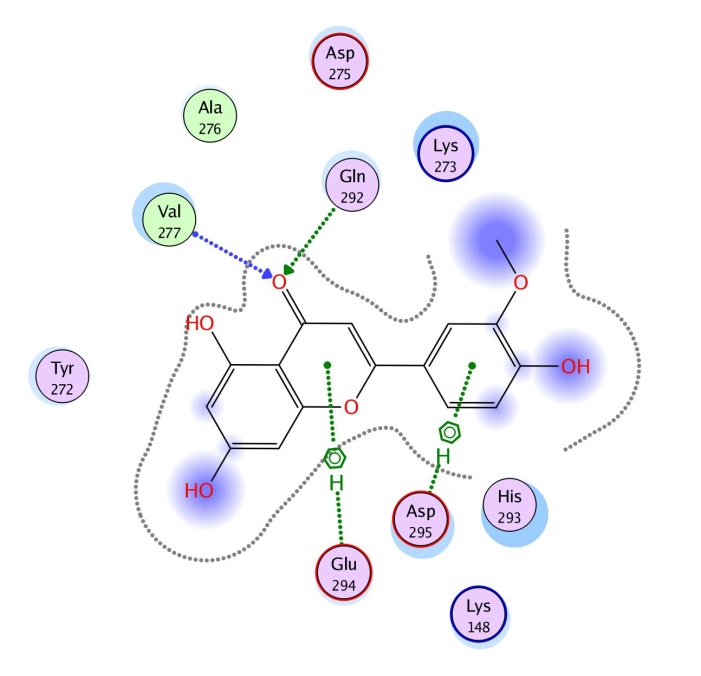** | **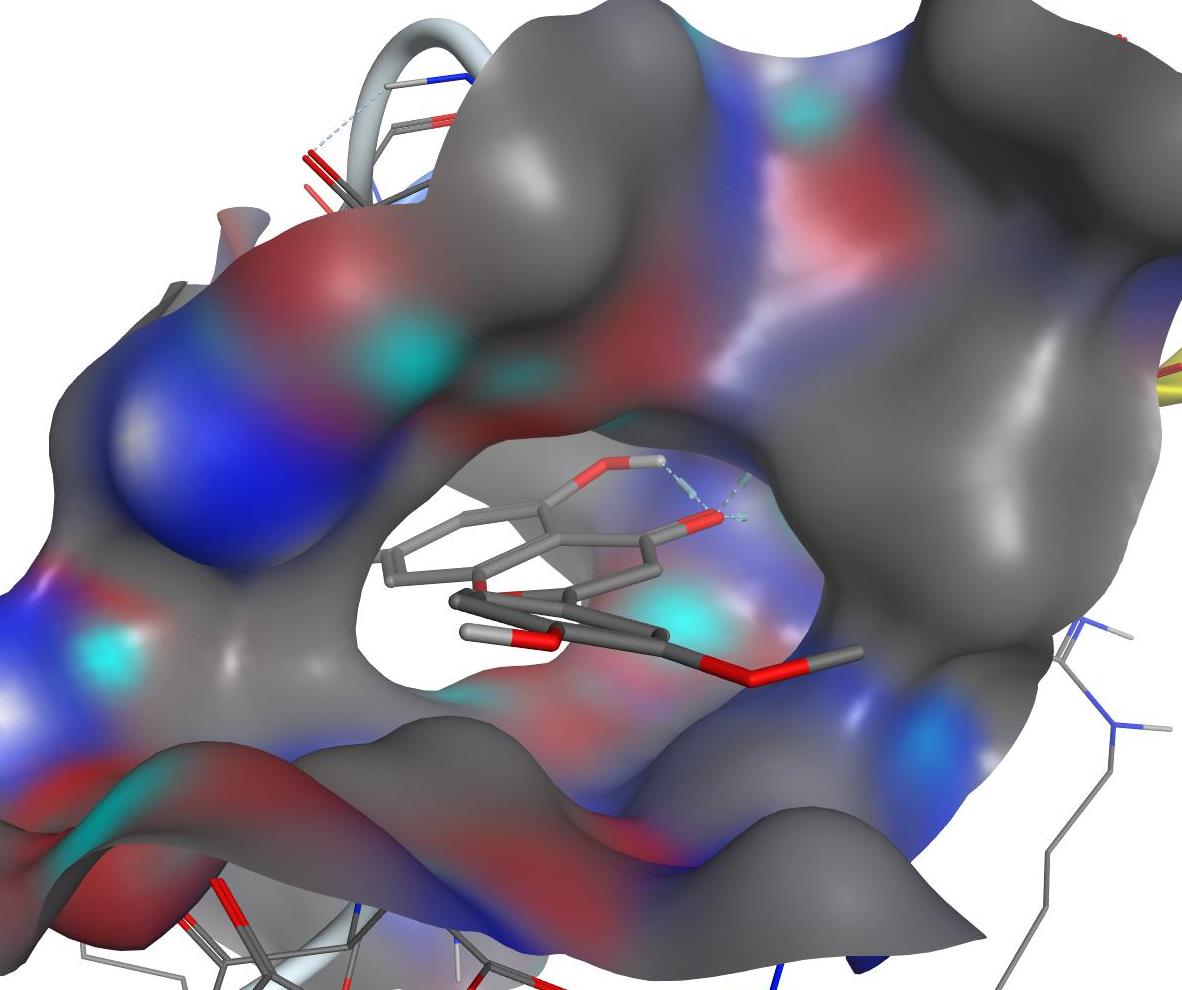** |
| --- | --- |

**Figure S23: 2D and 3D interactions of chrysoeriol with PBP2a allosteric site**

| **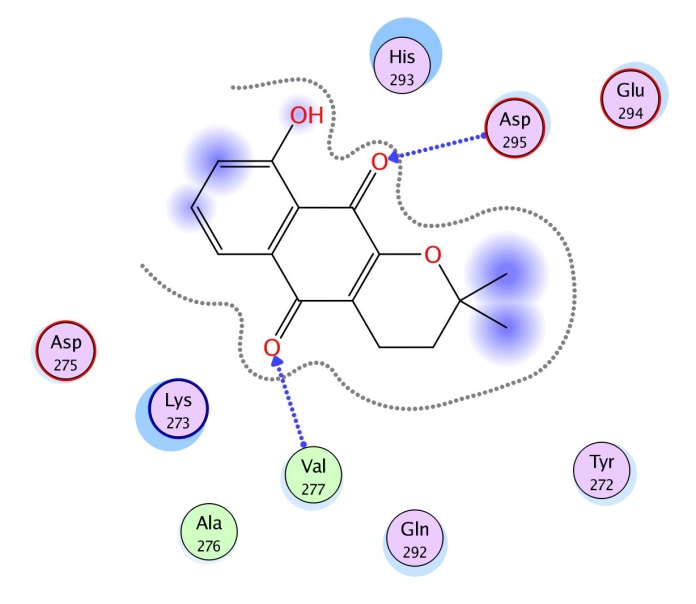** | **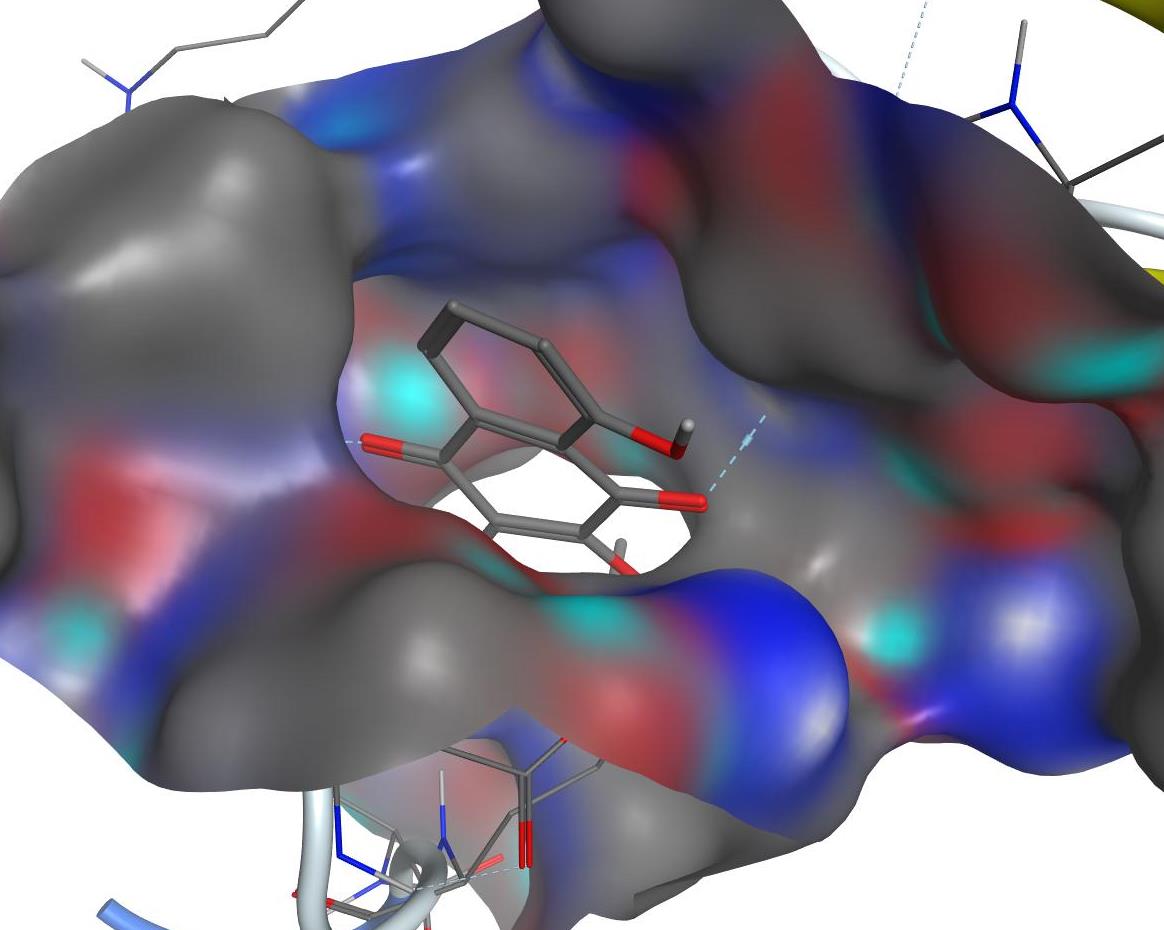** |
| --- | --- |

**Figure S24: 2D and 3D interactions of α-lapachone with PBP2a allosteric site**

**TABLE S1:** Phytochemicals that do not follow the Ro5

| Sr. No. | Name &  PubChem  CID | 2D Structure | S  (Kcal/mol) | RMSD  (Å) | SITE |
| --- | --- | --- | --- | --- | --- |
| 1 | Asphodoside D  CS ID 102144710 | 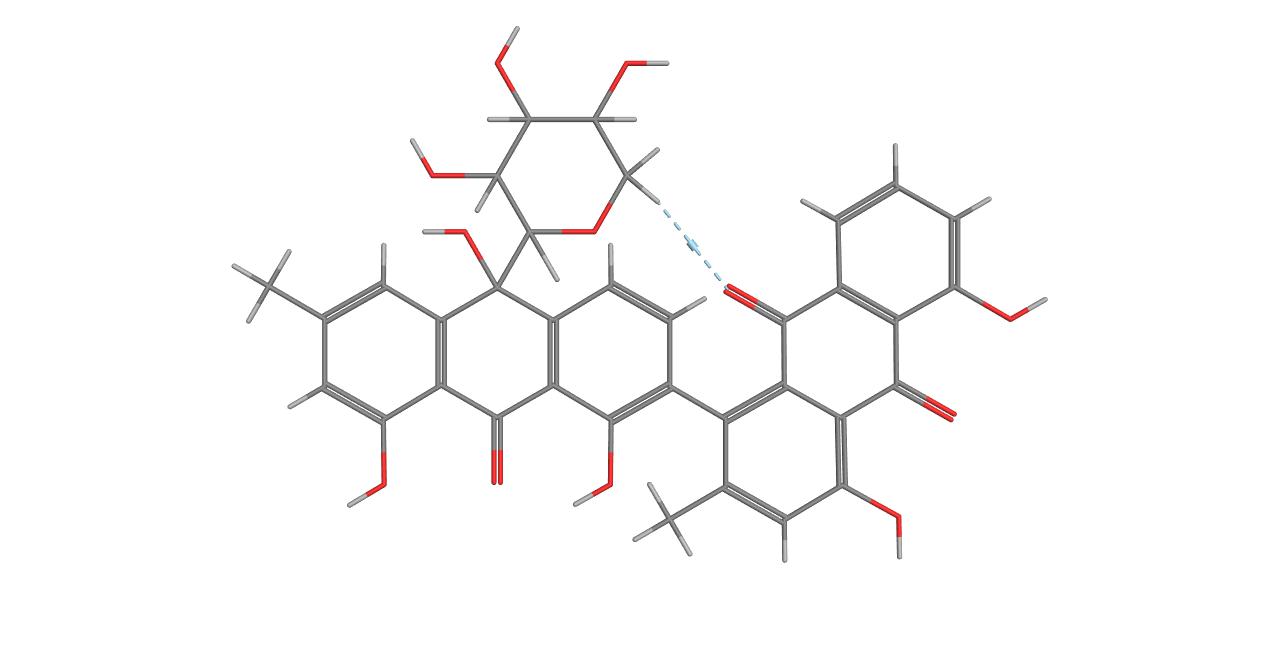 | -22.685 | 2.30 | 1 |
| 2 | Bacopasaponin A  CS ID 101995276 | 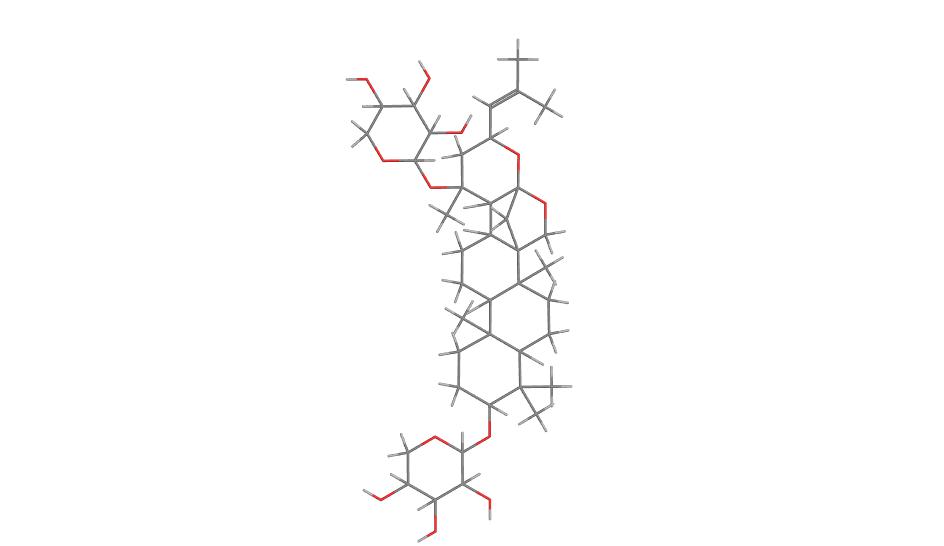 | -21.482 | 1.29 | 1 |
| 3 | Diosmin 5281613 | 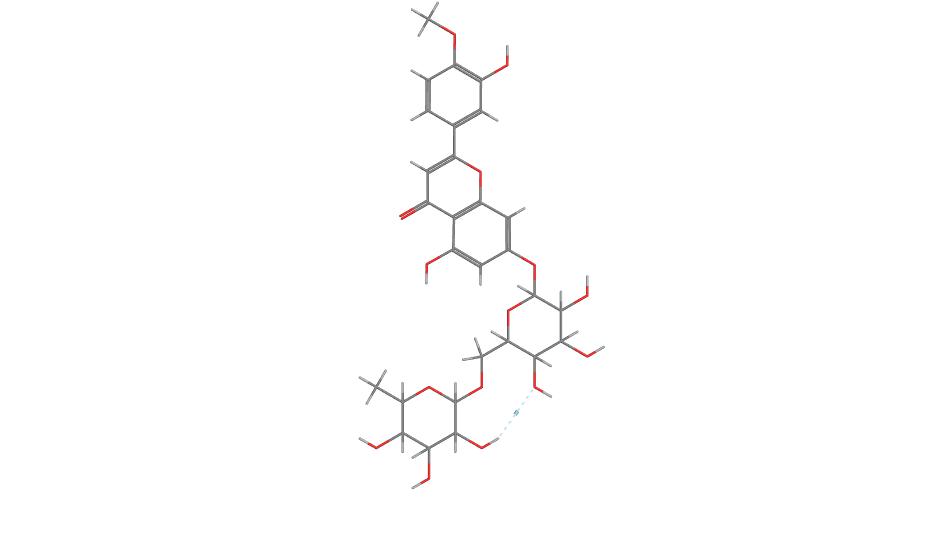 | -19.575 | 1.62 | 1 |
| 4 | Rutin 5280805 | 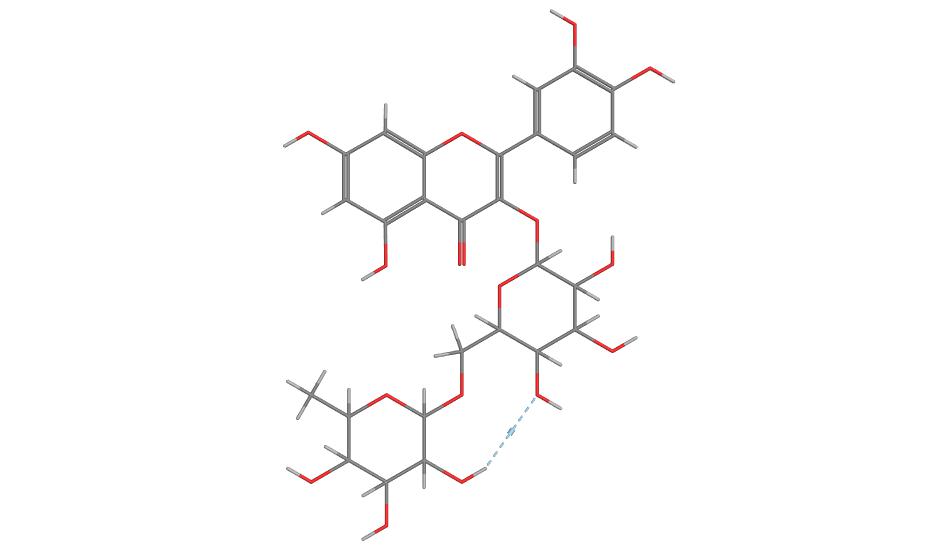 | -17.376 | 1.99 | 1 & 3 |

Note: CS ID= ChemSpider database ID, S= binding affinity of ligand with PBP2a, RMSD= root mean square deviation score of ligand pose in active site of PBP2a, SITE= active site of PBP2a where ligand is bounded

**TABLE S2:** ADME Properties of phytochemicals that do not follow Ro5

| Sr. No. | Name | MW | #RB | #HBA | #HBD | TPSA | Consensus Log P | GI absorption | Lipinski  #violations | Score | Lipinski Test |
| --- | --- | --- | --- | --- | --- | --- | --- | --- | --- | --- | --- |
| 1 | asphodoside D | 887.06 | 8 | 17 | 10 | 266.91 | 0.29 | Low | 3 | -22.685 | Pass |
| 2 | Bacopasaponin A | 736.93 | 6 | 12 | 6 | 176.76 | 2.95 | Low | 3 | -21.482 | Pass |
| 3 | Diosmin | 608.54 | 7 | 15 | 8 | 238.2 | -0.52 | Low | 3 | -19.575 | Pass |
| 4 | Rutin | 610.52 | 6 | 16 | 10 | 269.43 | -1.51 | Low | 3 | -17.376 | Pass |

*Note:* MW=Molecular weight, HBA=Hydrogen Bond Accepter, HBD=Hydrogen Bond Donor, TPSA=Topological Polar Surface Area, Logp=Lippophilicity Coefficient.

**TABLE S3:** Toxicities and bioactivities of phytochemicals that do not follow Ro5

| Names | Toxicities predicted by DataWarrior software | | | | Bioactivities predicted by Molinspiration database | | | | | |
| --- | --- | --- | --- | --- | --- | --- | --- | --- | --- | --- |
|  | Mutagenic | Tumorigenic | Reproductivity effects | irritant | GPCR Ligand | Ion channel inhibitor | Kinase inhibitor | Nuclear receptor ligand | Protease inhibitor | Enzyme inhibitor |
| asphodoside D | None | none | none | none | 0.28 | -0.14 | -0.01 | -0.2 | 0.5 | 0.28 |
| Bacopasaponin A | None | none | none | none | 0.27 | 0.37 | 0.06 | -0.01 | 0.19 | 0.28 |
| Diosmin | None | none | none | none | -0.05 | -0.52 | -0.14 | -0.23 | -0.07 | 0.12 |
| Rutin | None | none | none | none | -0.05 | -0.52 | -0.14 | -0.23 | -0.07 | 0.12 |

*Note:* Mutagenic= cause damage in genetic material, Tumorigenic= Tumor causing, Reproductive Effective= Interfere in normal reproduction, Irritant= causes slight inflammation or other discomfort to the body, if bioactivity score of phytochemical is (>0) then it is active, if (-5.0-0.0) then moderately active, if (< -5.0) then inactive.

**TABLE S4:** Name, PubChem ID, 2D structure, binding affinities and binding site of Penicillins

| Sr. No. | Names &  PubChem CID | 2D Structure | S (kcal/mol) | RMSD (Å) | SITE |
| --- | --- | --- | --- | --- | --- |
| 1 | Ticarcillin  36921 | 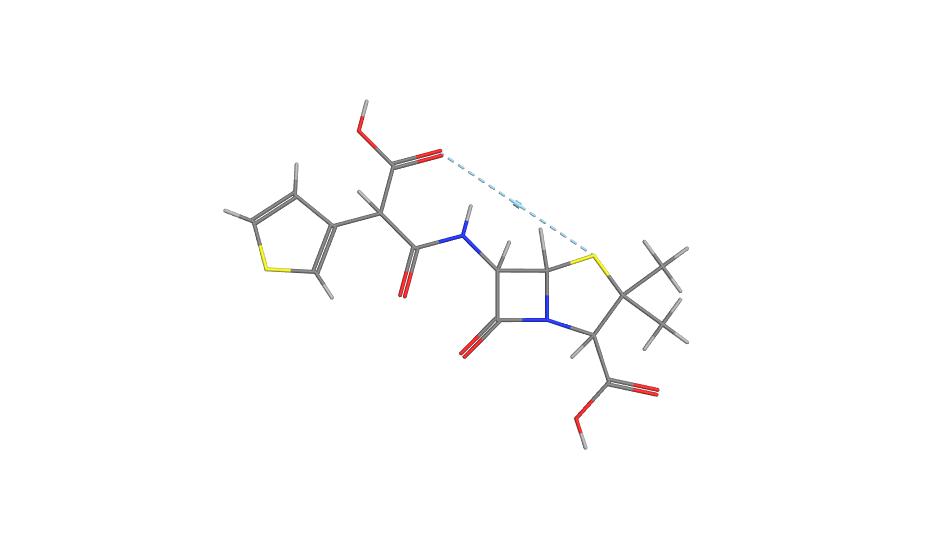 | -13.753 | 1.80 | 1 |
| 2 | Penicillin V  6869 | 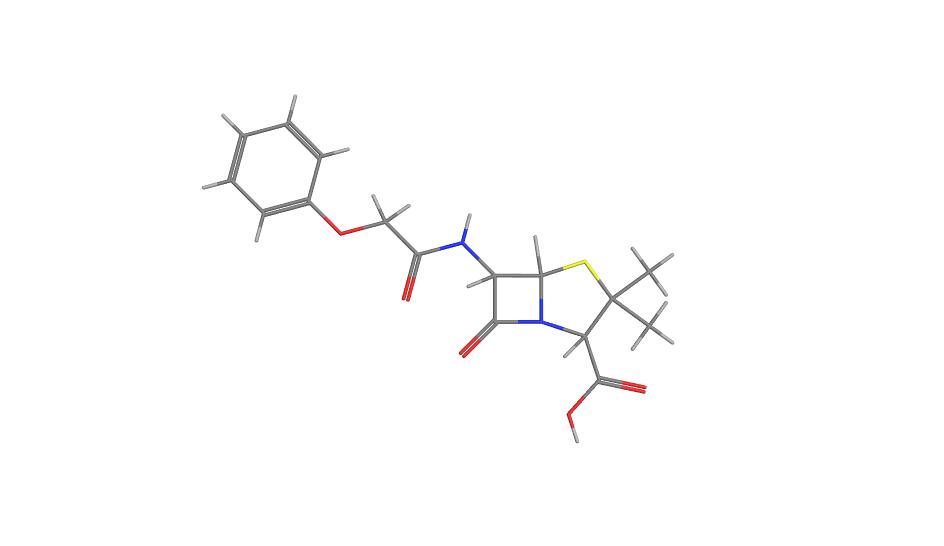 | -12.966 | 1.27 | 1 |
| 3 | Piperacillin  43672 | 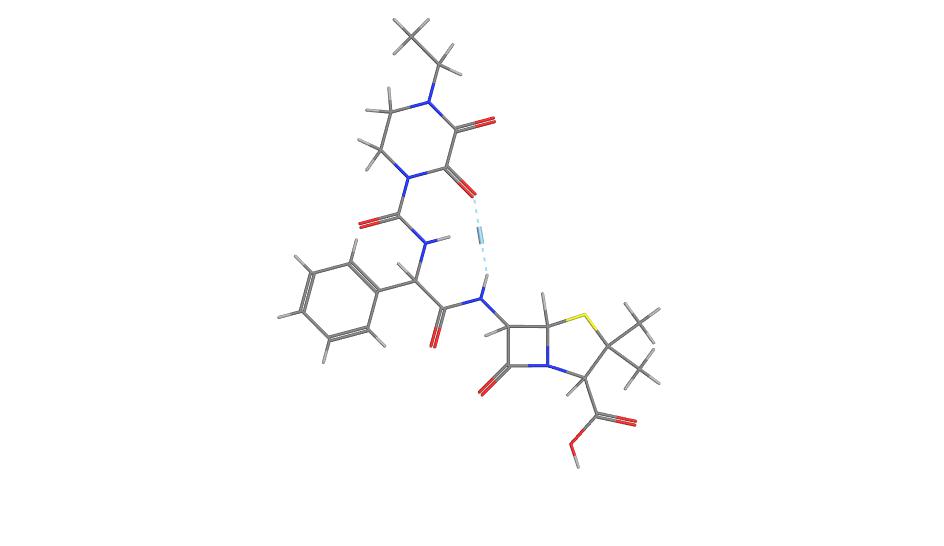 | -12.925 | 2.16 | 1 & 3 |
| 4 | Carbenicillin  20824 | 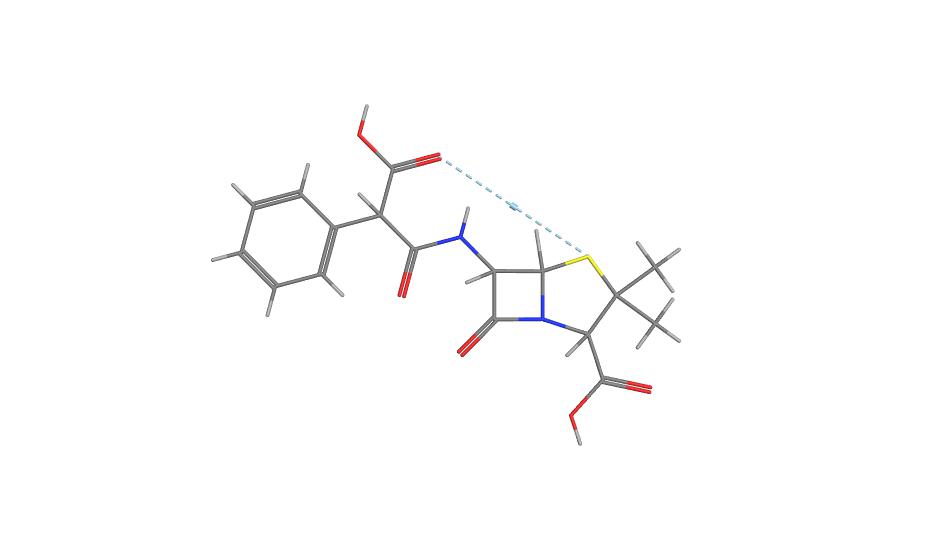 | -12.543 | 1.05 | 1 |
| ­5 | Dicloxacillin  18381 | 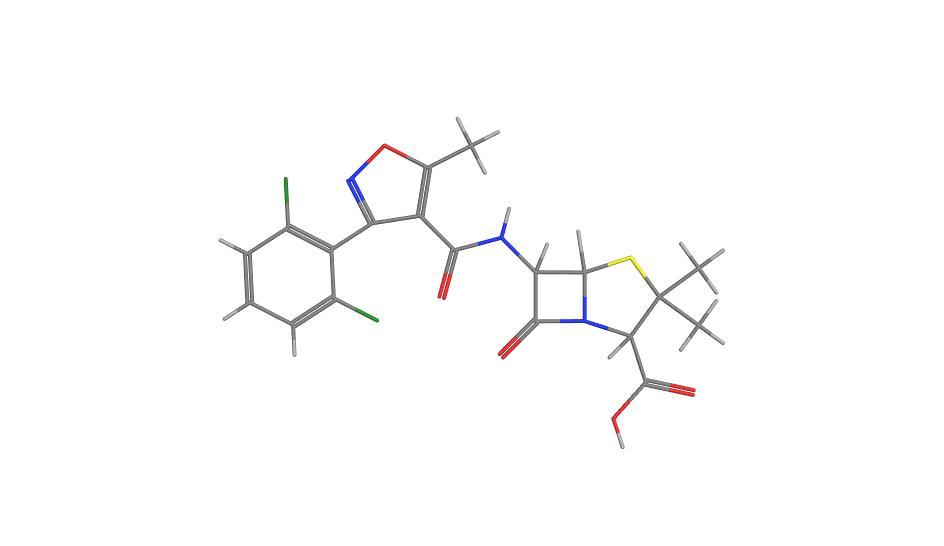 | -12.280 | 2.78 | 1 |
| 6 | Oxacillin  6196 | 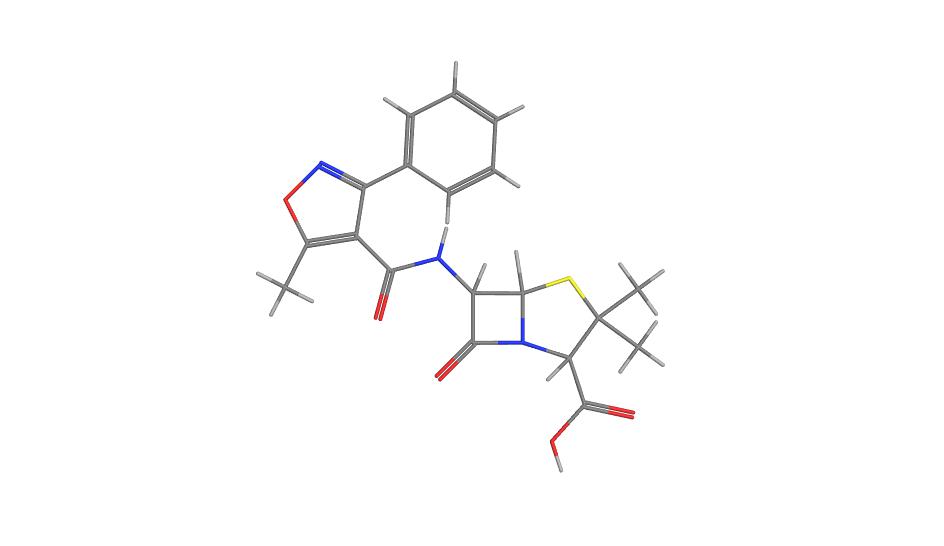 | -12.238 | 1.65 | 1 |
| 7 | Cloxacillin  6098 | 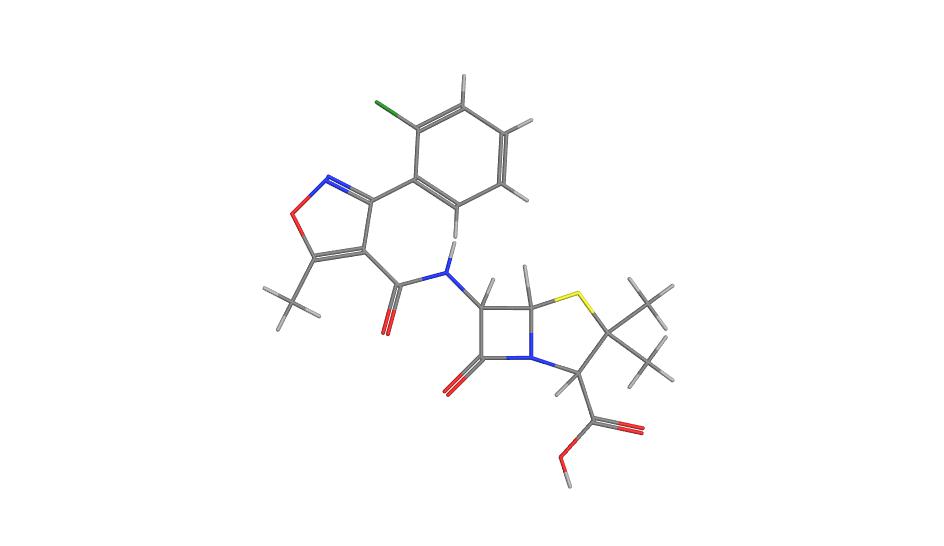 | -11.618 | 1.27 | 1 |
| 8 | Nafcillin  8982 | 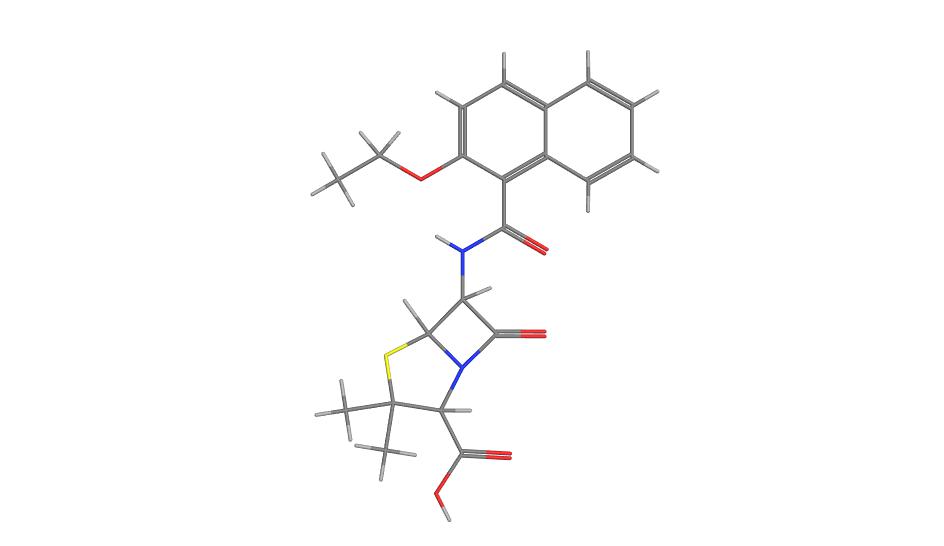 | -11.488 | 1.12 | 1 |
| 9 | Penicillin G  5904 | 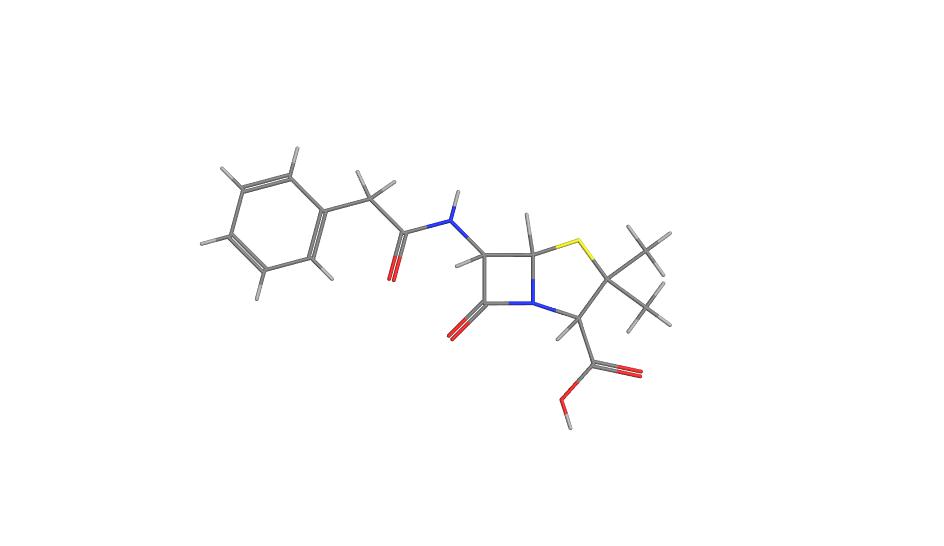 | -11.408 | 1.07 | 1 |
| 10 | Methicillin  6087 | 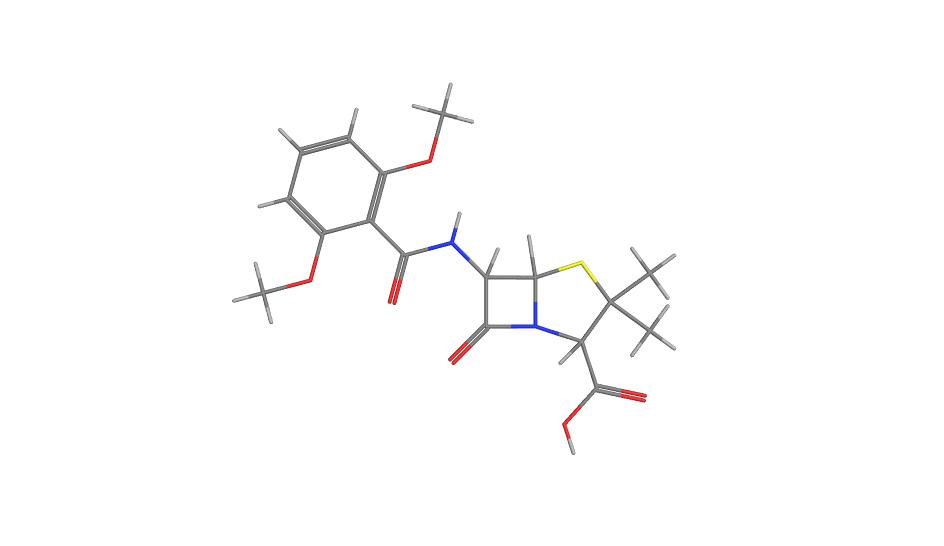 | -11.241 | 1.40 | 1 |
| 11 | Amoxicillin  33613 | 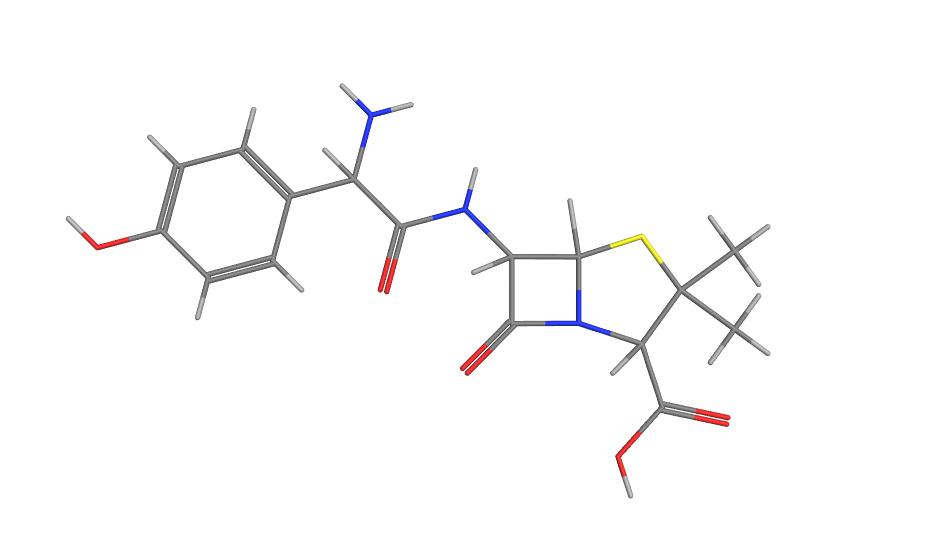 | -11.241 | 1.40 | 1 |
| 12 | Ampicillin  6249 | 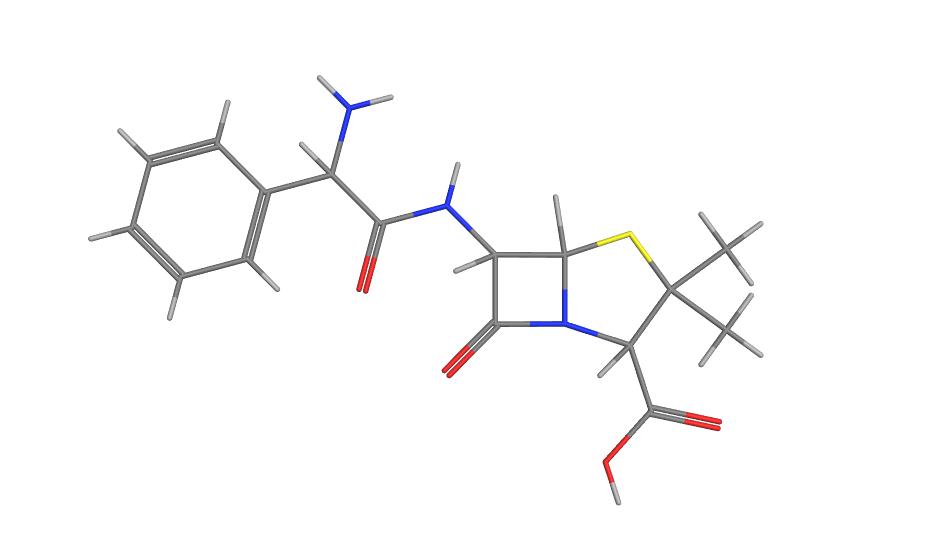 | -10.361 | 2.52 | 1 |

Note: S= binding affinity of ligand with PBP2a, RMSD= root mean square deviation score of ligand pose in active site of PBP2a, SITE= active site of PBP2a where ligand is bounded

**TABLE S5:** ADME properties of penicillins

| Sr. No. | Name | MW | #RB | #HBA | #HBD | TPSA | Consensus Log P | GI absorption | Lipinski  #violations | Score | Lipinski Test |
| --- | --- | --- | --- | --- | --- | --- | --- | --- | --- | --- | --- |
| 1 | Ticarcillin | 384.43 | 6 | 6 | 3 | 177.55 | 0.63 | Low | 0 | -13.753 | Pass |
| 2 | Penicillin V | 350.39 | 6 | 5 | 2 | 121.24 | 1.15 | High | 0 | -12.966 | Pass |
| 3 | Piperacillin | 517.55 | 9 | 7 | 3 | 181.73 | -0.16 | Low | 2 | -12.9247 | Fail |
| 4 | Carbenicillin | 378.4 | 6 | 6 | 3 | 149.31 | 0.69 | Low | 0 | -12.5433 | Pass |
| 5 | Dicloxacillin | 470.33 | 5 | 6 | 2 | 138.04 | 2.72 | Low | 0 | -12.2802 | Pass |
| 6 | Oxacillin | 401.44 | 5 | 6 | 2 | 138.04 | 1.85 | High | 0 | -12.2377 | Pass |
| 7 | Cloxacillin | 435.88 | 5 | 6 | 2 | 138.04 | 2.22 | High | 0 | -11.6175 | Pass |
| 8 | Nafcillin | 414.47 | 6 | 5 | 2 | 121.24 | 2.35 | High | 0 | -11.4876 | Pass |
| 9 | Penicillin G | 334.39 | 5 | 4 | 2 | 112.01 | 1.3 | High | 0 | -11.4084 | Pass |
| 10 | Methicillin | 380.42 | 6 | 6 | 2 | 130.47 | 1.16 | High | 0 | -11.2409 | Pass |
| 11 | Amoxicillin | 365.4 | 5 | 6 | 4 | 158.26 | -0.39 | Low | 0 | -11.2395 | Pass |
| 12 | Ampicillin | 349.4 | 5 | 5 | 3 | 138.03 | 0.08 | Low | 0 | -10.3608 | Pass |

Note: MW=Molecular weight, HBA=Hydrogen Bond Accepter, HBD=Hydrogen Bond Donor, TPSA=Topological Polar Surface Area, Logp=Lippophilicity Coefficient.

**TABLE S6:** Toxicities of penicillins predicted by DataWarrior Software

| S.NO | Name | Mutagenic | Tumorigenic | Reproductive Effective | Irritant |
| --- | --- | --- | --- | --- | --- |
| 1 | Ticarcillin | none | none | none | none |
| 2 | Penicillin V | none | **High** | none | none |
| 3 | Piperacillin | none | none | none | none |
| 4 | Carbenicillin | none | none | none | none |
| 5 | Dicloxacillin | **high** | none | none | none |
| 6 | Oxacillin | **high** | none | none | none |
| 7 | Cloxacillin | **high** | none | none | none |
| 8 | Nafcillin | **high** | **High** | none | none |
| 9 | Penicillin G | **high** | **High** | none | none |
| 10 | Methicillin | none | none | none | none |
| 11 | Amoxicillin | none | none | none | none |
| 12 | Ampicillin | none | none | none | none |

*Note:* Mutagenic= cause damage in genetic material, Tumorigenic= tumor causing, Reproductive Effective= interfere in normal reproduction, Irritant= causes slight inflammation or other discomfort to the body

**TABLE S7:** Name, PubChem ID, 2D structure, score of General Antibiotics and binding SITE of PBP2a

| Sr. No. | Names & PubChem CID | 2D Structures | S (Kcal/mol) | RMSD (Å) | SITE |
| --- | --- | --- | --- | --- | --- |
| 1 | Amikacin  37768 | 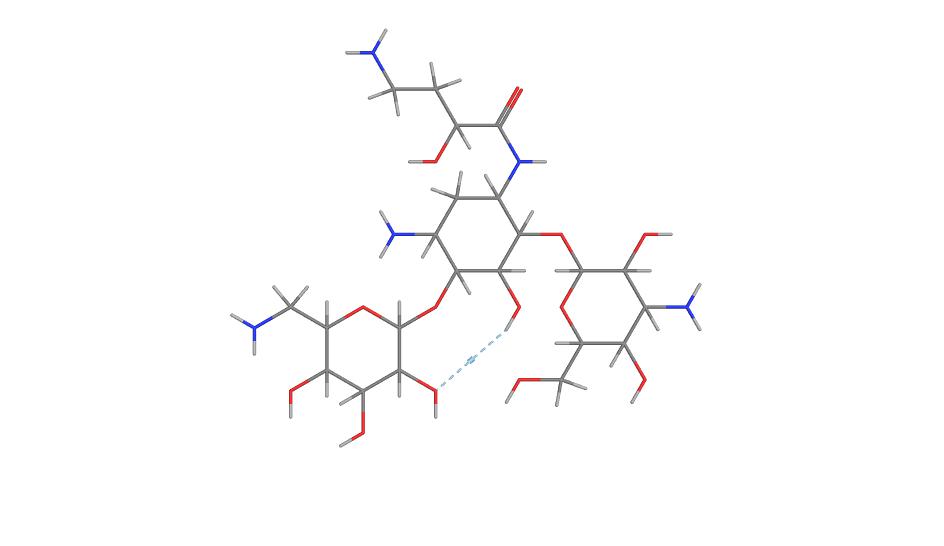 | -18.624 | 2.18 | 1 |
| 2 | Gentamicin  3467 | 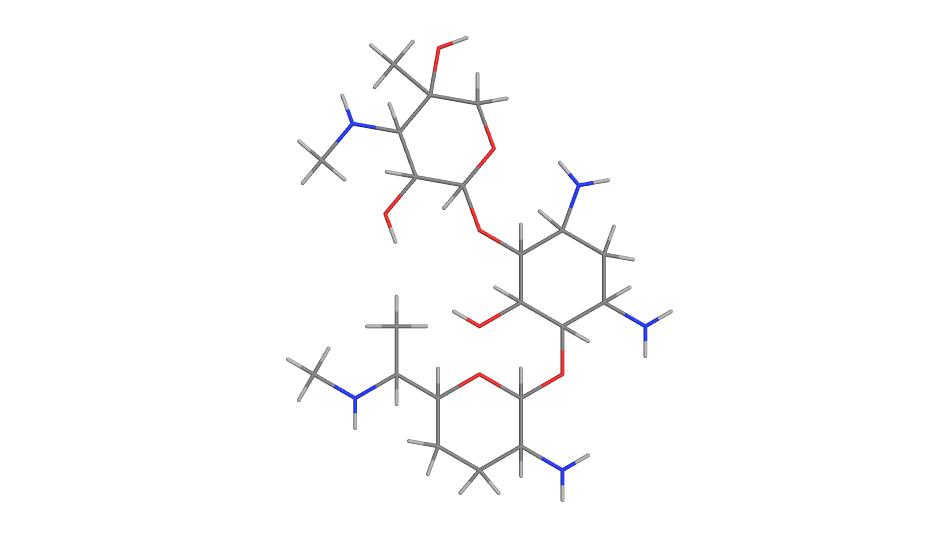 | -18.341 | 2.04 | 1 |
| 3 | Vancomycin  14969 | 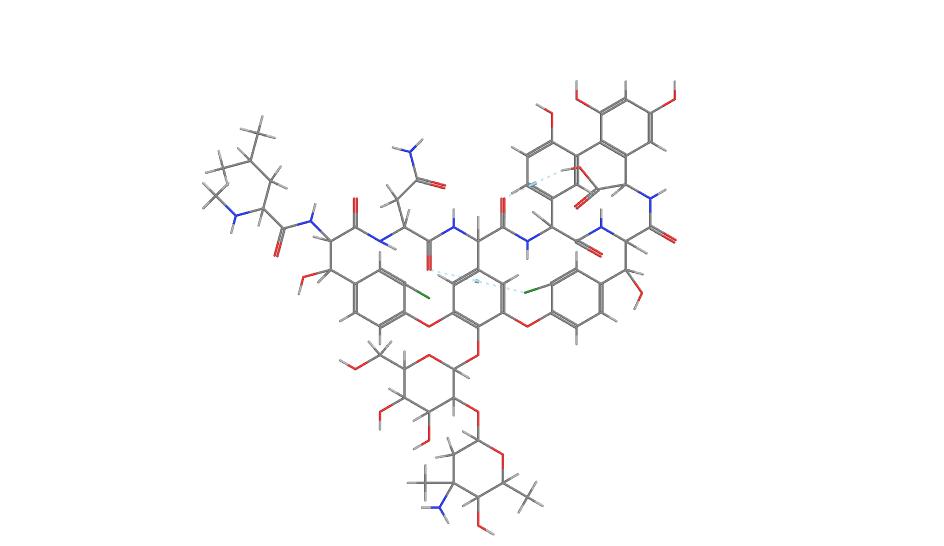 | -16.878 | 2.80 | 1 & 5 |
| 4 | Oxytetracycline  54675779 | 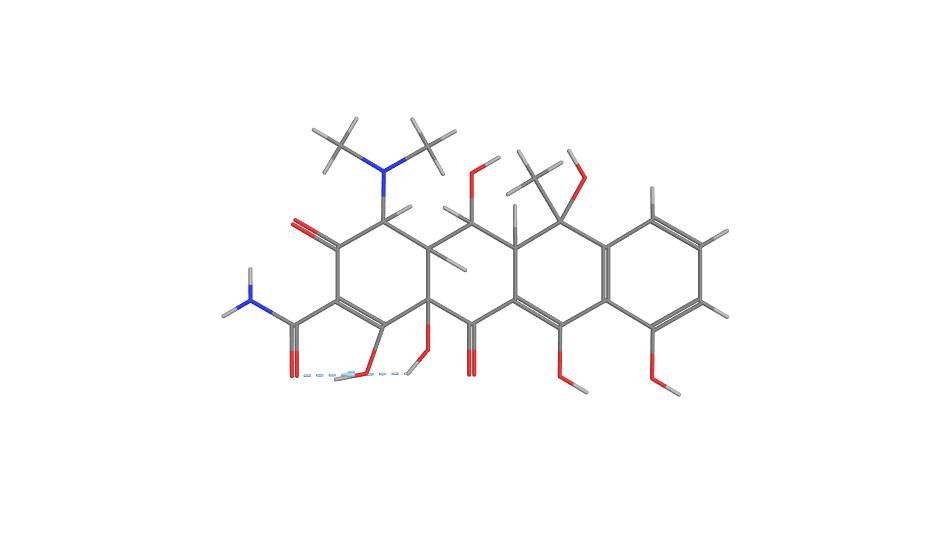 | -15.083 | 1.48 | 1 |
| 5 | Cefoxitin  441199 | 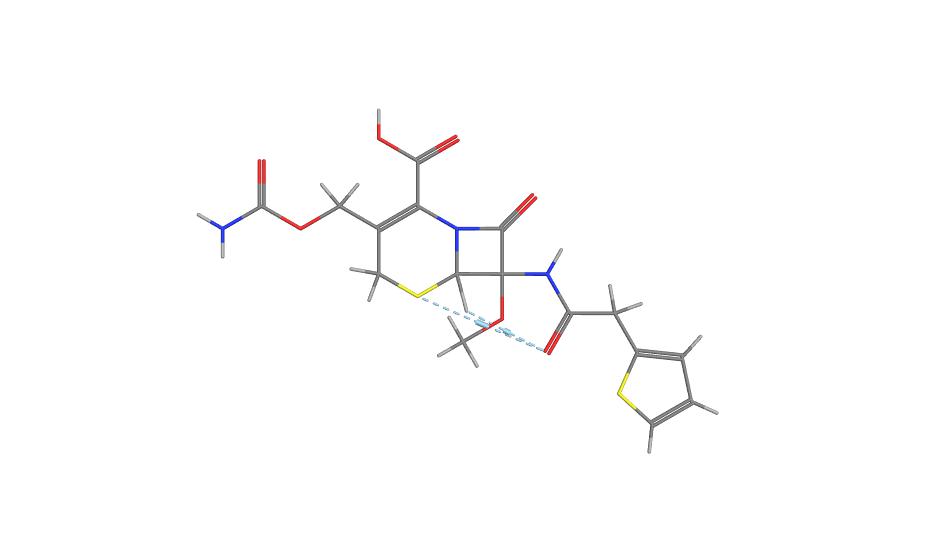 | -13.070 | 2.22 | 1 |
| 6 | Oxacillin  6196 | 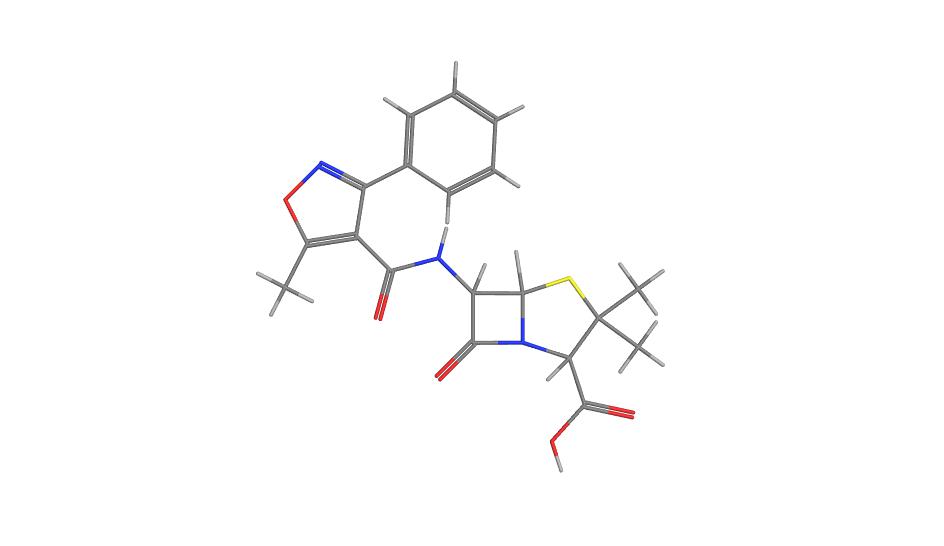 | -12.288 | 0.98 | 1 |
| 7 | Ceftobiprole  135413542 | 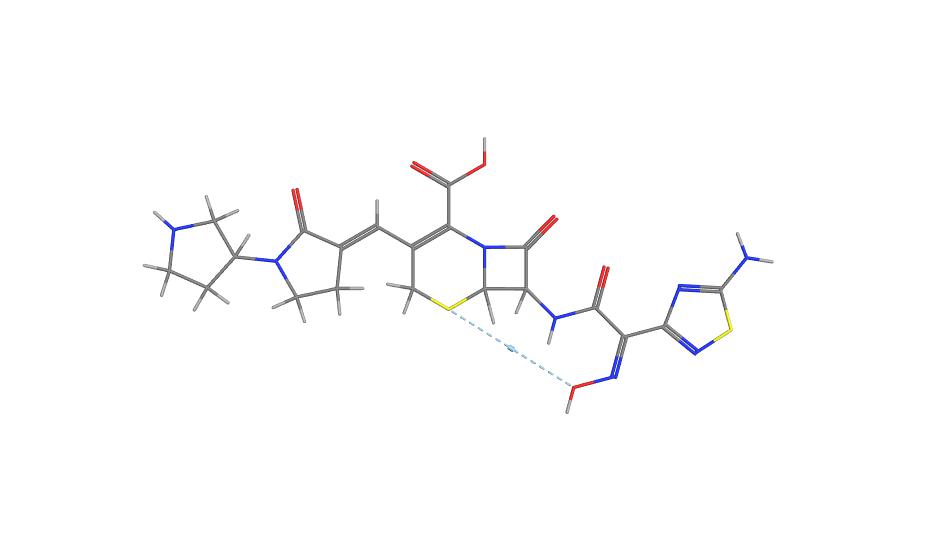 | -12.110 | 1.73 | 3 |
| 8 | Chloramphenicol  5959 | 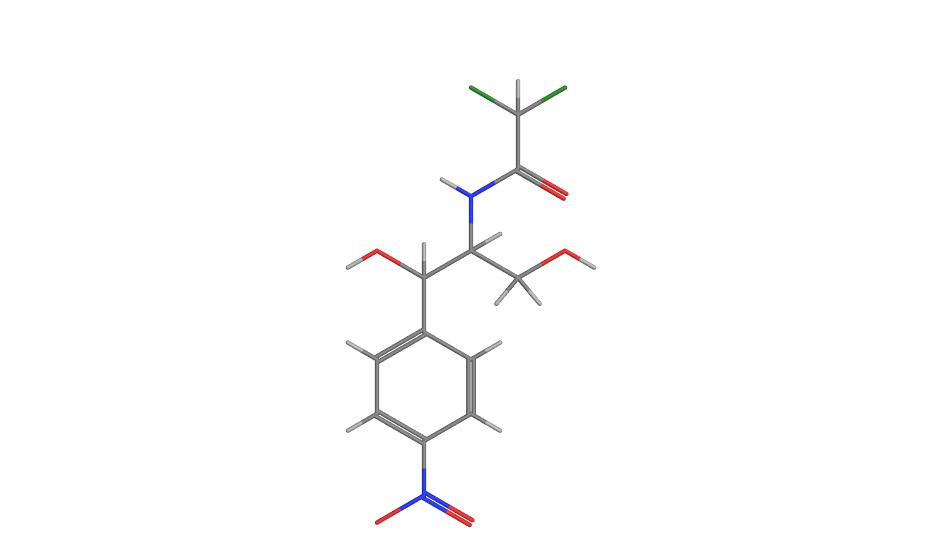 | -12.080 | 1.10 | 1 |
| 9 | Trimethoprim-Sulphmethoxazole  5578 | 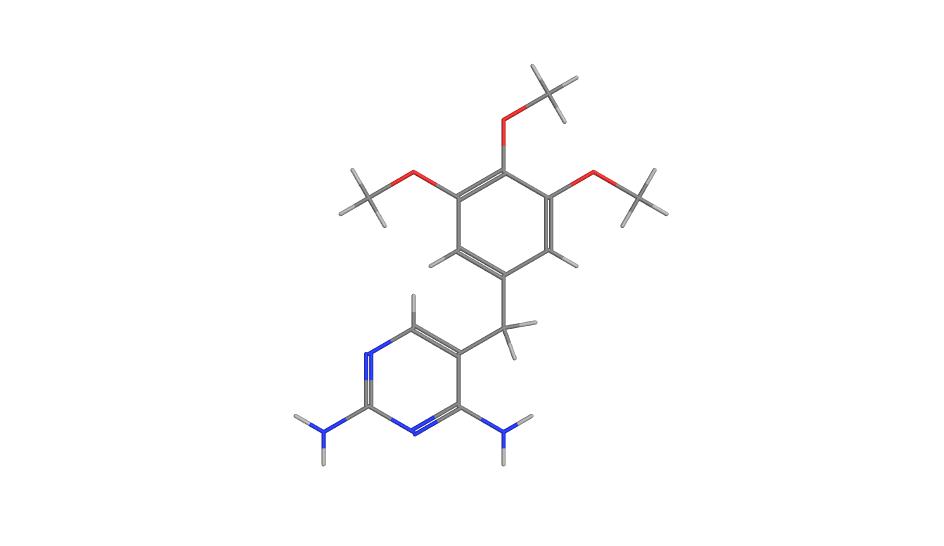 | -12.02 | 0.99 | 1 |
| 10 | Ampicillin  6249 | 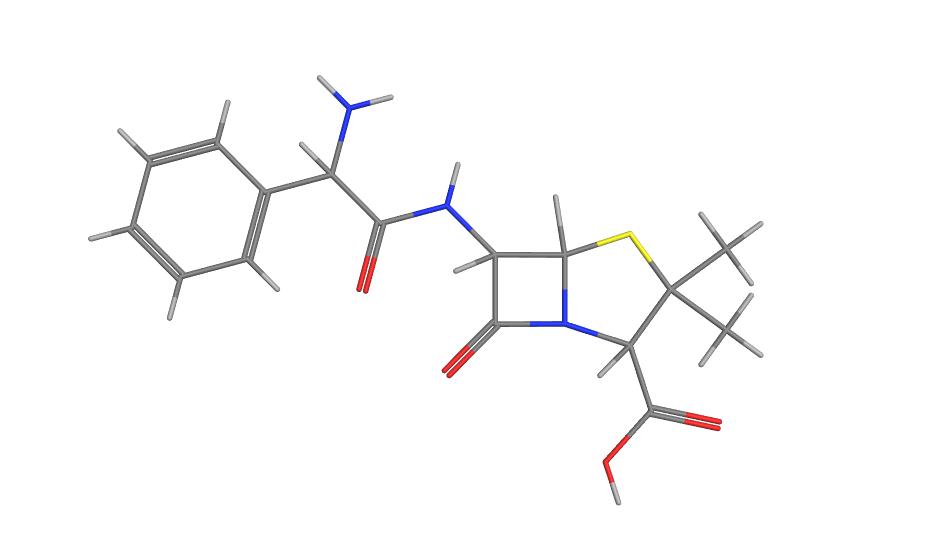 | -11.520 | 1.44 | 1 |
| 11 | Ciprofloxacin  2764 | 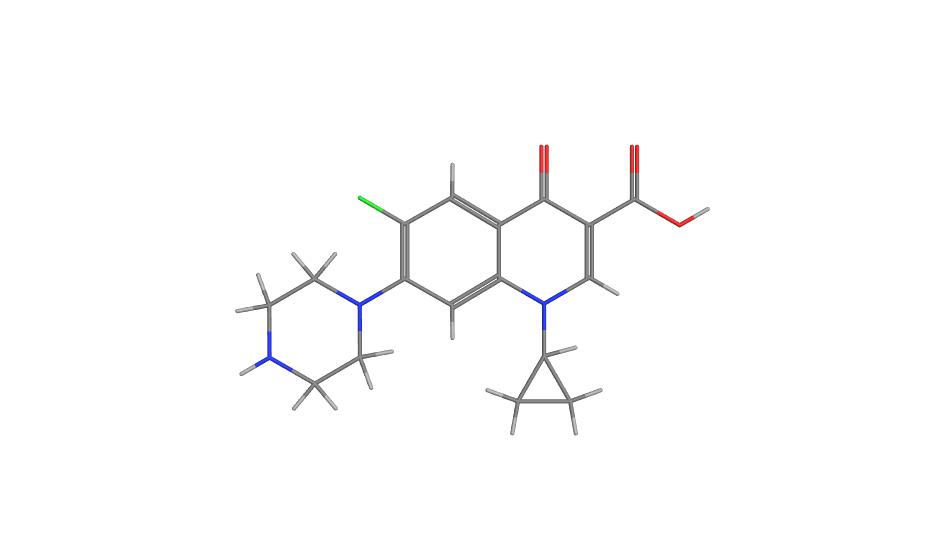 | -10.887 | 1.16 | 1 |
| 12 | Fusidic Acid  3000226 | 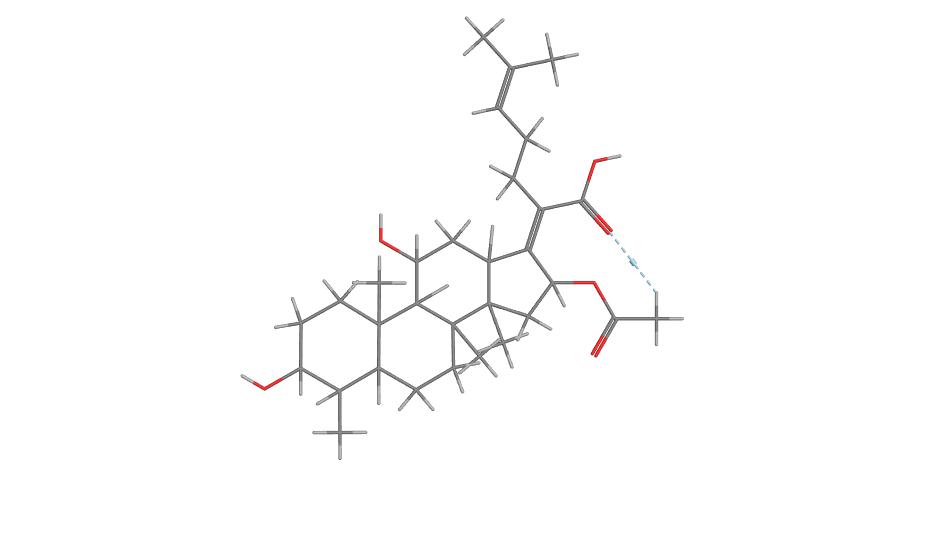 | -10.017 | 0.85 | 1 |

*Note:* S= binding affinity of ligand with PBP2a, RMSD= root mean square deviation score of ligand pose in active site of PBP2a, SITE= active site of PBP2a where ligand is bounded

**TABLE S8:** Drug Likeliness Properties of General Antibiotics

| Sr. No. | Name | MW | #RB | #HBA | #HBD | TPSA | Consensus Log P | GI absorption | Lipinski #violations | S | Lipinski’s Test |
| --- | --- | --- | --- | --- | --- | --- | --- | --- | --- | --- | --- |
| 1 | Amikacin | 588.63 | 11 | 14 | 13 | 336.8 | -8.55 | Low | 3 | -18.624 | Fail |
| 2 | Gentamicin | 481.63 | 7 | 7 | 7 | 216.58 | -7.04 | Low | 2 | -18.341 | Fail |
| 3 | Vancomycin | 1448.25 | 14 | 26 | 18 | 533.32 | -2.78 | Low | 3 | -16.878 | Fail |
| 4 | Oxytetracycline | 459.43 | 2 | 10 | 6 | 204.68 | -1.11 | Low | 2 | -15.083 | Fail |
| 5 | Cefoxitin | 427.45 | 9 | 7 | 3 | 201.8 | 0.28 | Low | 0 | - 13.070 | Pass |
| 6 | Chloramphenicol | 323.13 | 7 | 5 | 3 | 115.38 | 0.53 | High | 0 | -12.080 | Pass |
| 7 | Trimethoprim-Sulphmethoxazole | 290.32 | 5 | 5 | 2 | 105.51 | 1.16 | High | 0 | -12.020 | Pass |
| 8 | Ciprofloxacin | 331.34 | 3 | 4 | 1 | 81.98 | -0.16 | High | 0 | -10.887 | Pass |
| 9 | Fusidic Acid | 515.7 | 6 | 6 | 2 | 106.89 | 4.5 | High | 1 | -10.017 | Pass |

*Note*: MW=Molecular weight, HBA=Hydrogen Bond Accepter, HBD=Hydrogen Bond Donor, TPSA=Topological Polar Surface Area, Logp=Lippophilicity Coefficient.

**TABLE S9:** Toxicities of general antibiotics Predicted by DataWarrior software

| Names | Mutagenic | Tumorigenic | Reproductive Effective | Irritant |
| --- | --- | --- | --- | --- |
| Amikacin | none | none | none | none |
| Gentamicin | none | none | none | none |
| Vancomycin | none | none | none | none |
| Oxytetracycline | none | none | **high** | none |
| Cefoxitin | none | none | none | none |
| Chloramphenicol | **high** | **high** | **high** | **high** |
| Trimethoprim-Sulphmethoxazole | none | none | none | none |
| Ciprofloxacin | none | none | none | none |
| Fusidic Acid | none | none | none | **high** |

*Note:* Mutagenic= cause damage in genetic material, Tumorigenic= tumor causing, reproductive Effective= interfere in normal reproduction, Irritant= causes slight inflammation or other discomfort to the body
